# Genetic connectivity and population structure of African savanna elephants (*Loxodonta africana*) in Tanzania

**DOI:** 10.1002/ece3.6728

**Published:** 2020-10-09

**Authors:** George G. Lohay, Thomas Casey Weathers, Anna B. Estes, Barbara C. McGrath, Douglas R. Cavener

**Affiliations:** ^1^ Biology Department The Pennsylvania State University University Park PA USA; ^2^ Ecosystem Science and Management The Pennsylvania State University University Park PA USA; ^3^ Environmental Studies Department Carleton College Northfield MN USA; ^4^ The Nelson Mandela African Institution of Science and Technology Arusha Tanzania

**Keywords:** connectivity, conservation genetics, elephants, fragmentation, gene flow, microsatellites, migratory corridors, mitochondrial DNA, Tanzania

## Abstract

Increasing human population growth, exurban development, and associated habitat fragmentation is accelerating the isolation of many natural areas and wildlife populations across the planet. In Tanzania, rapid and ongoing habitat conversion to agriculture has severed many of the country's former wildlife corridors between protected areas. To identify historically linked protected areas, we investigated the genetic structure and gene flow of African savanna elephants in Tanzania using microsatellite and mitochondrial DNA markers in 688 individuals sampled in 2015 and 2017. Our results indicate distinct population genetic structure within and between ecosystems across Tanzania, and reveal important priority areas for connectivity conservation. In northern Tanzania, elephants sampled from the Tarangire‐Manyara ecosystem appear marginally, yet significantly isolated from elephants sampled from the greater Serengeti ecosystem (mean *F*_ST_ = 0.03), where two distinct subpopulations were identified.Unexpectedly, elephants in the Lake Manyara region appear to be more closely related to those across the East African Rift wall in the Ngorongoro Conservation Area than they are to the neighboring Tarangire subpopulations. We concluded that the Rift wall has had a negligible influence on genetic differentiation up to this point, but differentiation may accelerate in the future because of ongoing loss of corridors in the area. Interestingly, relatively high genetic similarity was found between elephants in Tarangire and Ruaha although they are separated by >400 km. In southern Tanzania, there was little evidence of female‐mediated gene flow between Ruaha and Selous, probably due to the presence of the Udzungwa Mountains between them. Despite observing evidence of significant isolation, the populations of elephants we examined generally exhibited robust levels of allelic richness (mean *A*_R_ = 9.96), heterozygosity (mean *µH*_E_ = 0.73), and effective population sizes (mean *N*_e_ = 148). Our results may inform efforts to restore wildlife corridors between protected areas in Tanzania in order to facilitate gene flow for long‐term survival of elephants and other species.

## Graphical abstract

We used microsatellite loci and mtDNA to identify population structure of African elephants in Northern Tanzania. We identified two subpopulations: Serengeti elephants were genetically distinct from Tarangire Manyara. We also found that the Tarangire elephants showed higher genetic similarity with the Ruaha elephants than the Serengeti.

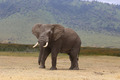

## 1 INTRODUCTION

**FIGURE 1:**
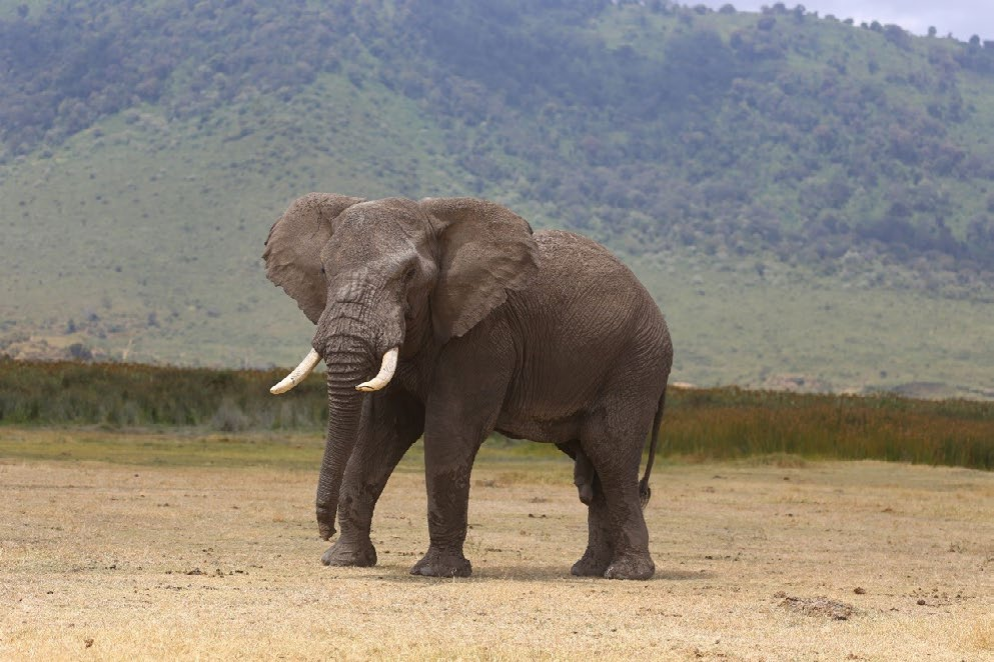
A photograph of a male elephant taken in Ngorongoro Conservation Area, one of our study areas. A male elephant in Ngorongoro Crater in Tanzania, a photograph taken in July 2017 (credit: George Lohay)

Habitat loss and fragmentation are a significant challenge in species conservation and maintaining biodiversity worldwide (Henle, Davies, Kleyer, Margules, & Settele, 2004). Human population growth near protected area (PA) boundaries is often higher than in comparable rural areas (Wittemyer, Elsen, Bean, Burton, & Brashares, 2008). Population growth often brings changes in land use for agriculture and settlements, which leads to loss of buffer zones adjacent to, and corridors connecting PAs. The isolation of PAs decreases their effective size, limits gene flow between populations, and leads to increased human‐wildlife conflicts. Elephant (*Loxodonta africana)* populations have been declining across Africa. Tanzania alone has lost over 60% (from 109,051 to 43,330) of its elephant population between 2009 and 2014 (TAWIRI, 2015). While poaching and illegal ivory trade are the most severe and immediate threats to African elephants (*referred to as* elephants), range and habitat fragmentation remain a significant long‐term threat to the species’ survival (CITES, 2014; Douglas‐Hamilton, 1987; Wittemyer et al., 2014). Habitat fragmentation mainly affects far‐ranging species, like the elephant. Elephants have extensive individual home ranges from 10 to 10,738 km^2^ (Douglas‐Hamilton, 1971; Leuthold & Sale, 1973; Lindeque & Lindeque, 1991; Thouless, 1995, 1996; Whyte, 1996), and they show high fidelity to their home ranges and corridors over multiple generations (Desai & Baskaran, 1996). Habitat loss and fragmentation resulting from human population growth and habitat conversion are of particular concern in Tanzania (Newmark, 2008) and threatens the connectivity of elephant populations that are becoming confined inside PAs. Riggio and Caro (2017) have shown that nearly a sixth of all the wildlife corridors identified in Tanzania in 2009 have potentially been separated by land conversion, and a third now pass across lands likely to be converted to human use in the near future. Without connectivity, many PAs in Tanzania are too small to be sustainable (Figure 1).

**FIGURE 2:**
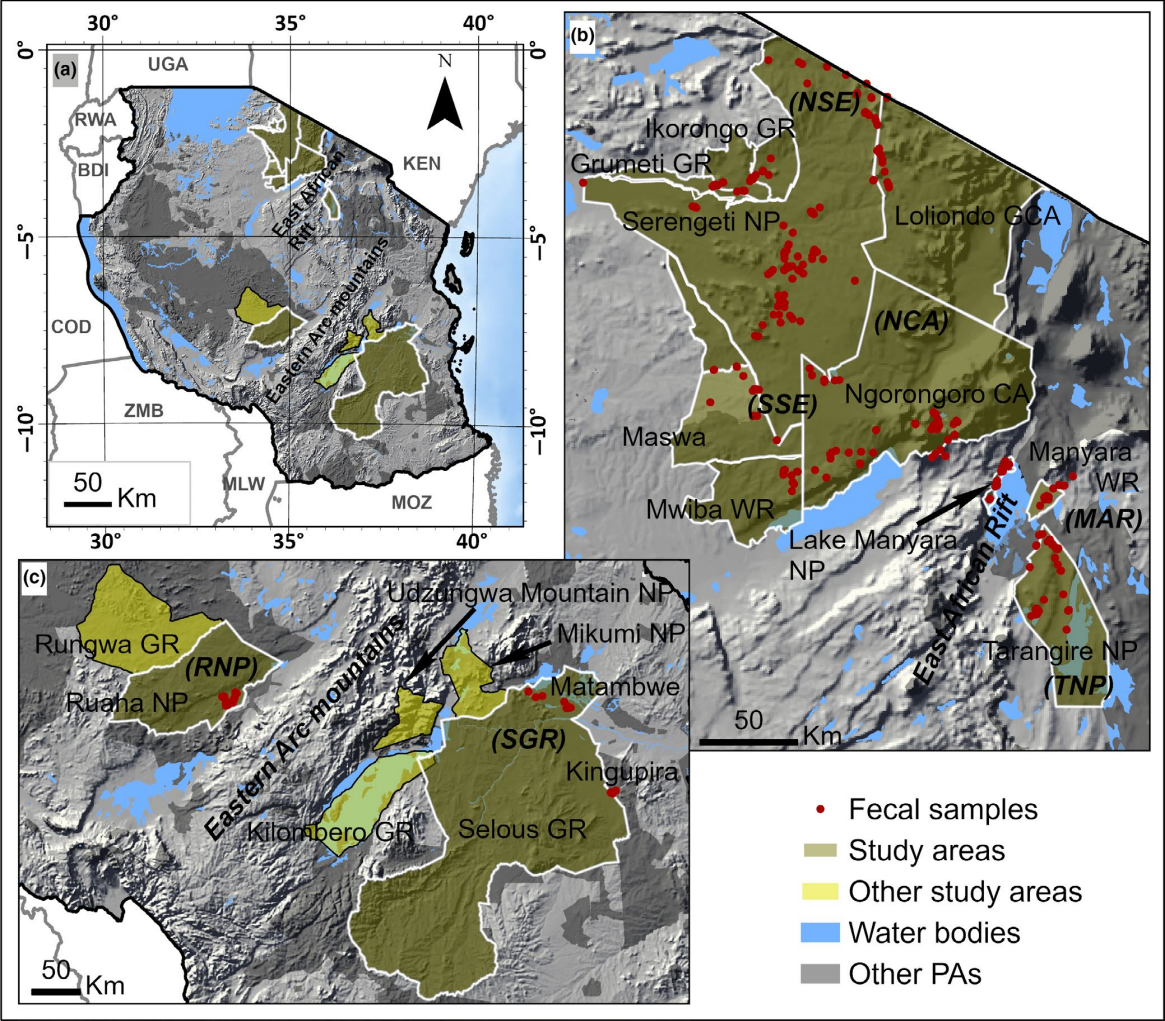
(a) Map of Tanzania showing four ecosystems where we collected elephant fecal samples. (n) Serengeti and Tarangire‐Manyara ecosystems comprising of northern Serengeti (NSE), southern Serengeti (SSE), Ngorongoro Conservation Area (NCA), Lake Manyara NP and Manyara Ranch (MAR), and Tarangire NP (TNP). The east African rift valley runs between these two ecosystems. **(C)** Ruaha and Selous ecosystems which are separated by the Eastern Arc Mountains. Red dots indicate areas where fecal samples were collected. NP, National Park; GCA, Game Controlled Area; GR, Game Reserve; WR, Wildlife Ranch and WM, Wildlife Management Area

The largest elephant populations in Tanzanian protected areas are found in four ecosystems: Ruaha, Selous, Serengeti, and Tarangire‐Manyara (Figure 2). All four ecosystems are important for biodiversity conservation in Tanzania. The Serengeti and Tarangire‐Manyara ecosystems are vital areas in Tanzania for the conservation of biodiversity. The East African Rift wall (500–1,000 m height) bounds the eastern edge of the Serengeti and the western edge of the Tarangire‐Manyara ecosystems (McNaughton & Campbell, 1991). However, outside these PAs, there is an increase in human population and the conversion of land to agriculture. For example, in the western Serengeti, human population growth between 1988 and 2002 was 3.5% per year, and the highest rates of agricultural conversion were close to the PA boundary (Estes, Kuemmerle, Kushnir, Radeloff, & Shugart, 2012). This growth is higher than the national average which is 2.7% per year. Human activities affect the movement of elephants within and between ecosystems by reducing the size of wildlife corridors or introducing connectivity barriers such as roads and human settlements. This limits the gene flow between the populations, threatening their long‐term sustainability. In the Tarangire‐Manyara ecosystem, expanding cultivation toward Tarangire National Park (TNP) has severely restricted wildlife movements to dispersal areas outside the park by blocking their migratory corridors. Unfortunately, there is an overlap between land suitable for agriculture, migratory wildlife corridors, and wet season dispersal areas (Msoffe et al., 2011). Thus, the rapidity of rangeland conversion to farming presents significant threats to wildlife conservation and disrupts the ecosystem viability (Lowe & Allendorf, 2010). Similarly, connectivity areas for the Tarangire‐Manyara ecosystem (hereafter, TME) and the Serengeti ecosystem (henceforth, SE) may not be viable in the long term because of its increasing isolation by agricultural settlements (Mwalyosi, 1991).

Female elephants are philopatric and remain with their natal herd (Archie et al., 2007), whereas males are ejected from the herd upon sexual maturity and subsequently facilitate gene flow between herds (Poole, 1987; Archie et al., 2007; Hollister‐Smith et al., 2007). Male‐mediated dispersal has been documented among elephant populations in both Uganda (Nyakaana & Arctander, 1999) and Kenya (Okello et al., 2008). In both studies, there was a lack of congruence between mitochondrial DNA and nuclear‐based variation. Mitochondrial DNA data showed significant differentiation, whereas nuclear data showed low genetic subdivision between populations (Nyakaana & Arctander, 1999; Okello et al., 2008).

Ultimately, habitat and connectivity loss, gene flow mediated by elephant behavioral patterns, and a complicated history of episodic population dynamics interact to influence current elephant population genetic structure. For example, Serengeti elephants, like most elephant populations across Africa, have experienced several intense poaching episodes over the past 150 years. In the mid‐nineteenth century, there were more than 4,000 elephants in the Serengeti (Sinclair, 2012). However, by 1890 elephants were virtually eliminated from the Serengeti and remained very low for six decades as a result of game hunting and the ivory trade (Sinclair, 2012). In 1950 after the Serengeti was gazetted as a protected area, the elephant population increased to about 3,000 in 1975. This increase was followed by heavy poaching across the continent that caused a decline to only 400 elephants in Serengeti in 1986 (Sinclair, 2012). The international ban of the ivory trade by the Convention on International Trade of Endangered Species (CITES) in 1989 resulted in an increase of the elephant population to about 6,000 in 2014 (TAWIRI, 2015). It has been hypothesized that after heavy poaching, elephant recolonization in the Serengeti in 1950s came from the north and south of Serengeti (Sinclair, 2012; Watson & Bell, 1969). Analysis of elephant genetic structure within and between ecosystems in Tanzania can therefore help elucidate historical founder events, recent connectivity between populations, and consequently indicate priority areas to target connectivity conservation between important protected areas.

In Tanzania, previous studies (Ahlering et al., 2012; Ishida, Georgiadis, Hondo, & Roca, 2013) suggested that the East African or Gregory Rift Valley separated the distribution of the elephant mitochondrial DNA haplotypes (i.e., east and west of the Rift Valley which represents the Serengeti and Tarangire‐Manyara ecosystems, respectively). However, these studies were based on small sample sizes and did not cover all protected areas within these ecosystems. Furthermore, the genetic relationship between the elephants in northern and southern Tanzania is not known. For example, in recent years, the wildlife corridor between Ruaha and Tarangire National Parks has been blocked completely (Riggio & Caro, 2017) but we do not know the extent of previous gene flow between these two PAs which are about 450 km apart.

The Greater Ruaha and Selous ecosystems are essential areas for elephants in Tanzania, accounting for 54% of the national elephant population. Unfortunately, these areas were heavily impacted by poaching in recent years. In Selous, the elephant populations declined from 44,806 in 2009 to 15,217 in 2014, whereas in Ruaha the decline was from 34,664 in 2009 to 8,272 in 2014 (TAWIRI, 2015). To keep these populations healthy, and enable long‐term survival, it has been suggested that it is vital to maintain connectivity between these populations (Jones et al., 2012). There was previous movement of elephants between the Greater Ruaha and Selous ecosystem in 2009, through corridors that connect to Udzungwa and Mikumi National Park, (Nahonyo, 2009). However, these migratory routes, ranging and dispersal areas are threatened by habitat loss through the expansion of agriculture and human settlement, and poaching (Douglas‐Hamilton, Krink, & Vollrath, 2005; Galanti, Preatoni, Martinoli, Wauters, & Tosi, 2006; Wittemyer et al., 2008). On the migratory routes, poaching is more likely to occur than inside the protected areas (Nahonyo, 2009).

In recent years, the wildlife corridors between Selous Game Reserve and Udzungwa Mountains National Parks (Figure 2) have closed completely (Jones et al., 2012) and measures to restore corridors were proposed (Jones et al., 2012). However, restoration of corridors requires significant resources. For many long‐lived species, such as elephants, it is critically important to understand connectivity because the rate at which habitat is being changed is sufficiently rapid and an evolutionary response to the changes is unlikely (Balkenhol, Cushman, Waits, Storfer, 2016). There is a tendency to assume that elephants (and other wildlife) previously moved freely across the landscape and mated in a panmictic manner before habitat loss (Epps, Wasser, Keim, Mutayoba, & Brashares, 2013); however, there is no direct evidence for this.

Herein, we address six questions: (a) to what degree does the rift valley wall influence gene flow between the Serengeti and other metapopulations in northern Tanzania? (b) what is the genetic history of elephants that recolonized the Serengeti following poaching‐induced extirpation? (c) has genetic differentiation occurred within regions such as the Serengeti in the absence of barriers to migration and gene flow? (d) how has the recent blockage of the wildlife corridor between Tarangire and Ruaha impacted gene flow and genetic differentiation? (e) what is the extent of gene flow mediated by males versus females? (f) are Ruaha elephants genetically isolated from those of Selous and is the isolation recent and due to habitat loss or were the populations distinct even before the intensification of human activities?

## 2 MATERIALS AND METHODS

### 2.1 Description of study areas

To conduct a regional assessment of the genetic structure, we selected four major ecosystems in Tanzania that have the largest elephant populations. These are Serengeti and Tarangire‐Manyara in northern Tanzania and Ruaha and Selous ecosystems in central and southern Tanzania (Figure 2). We then compared our data with studies of other elephant populations in Africa.

#### 2.1.1 The Serengeti ecosystem

The Serengeti ecosystem (SE) is in the north‐east of Tanzania between 34°450–35°500E and 2°–3°200S and includes multiple levels of protected area (Ernest et al., 2012). The SE is comprised of the Serengeti National Park (SNP), Ikorongo Game Reserve (IGR), Grumeti Game Reserves (GGR), Maswa Game Reserve (MGR), Ngorongoro Conservation Area (NCA), Loliondo Game Controlled Area (LGCA), and Mwiba Wildlife Ranch (MWR) (Figure 2). The National Park offers the highest level of protection, excluding all human uses but for research and tourism. The Game Reserves allow trophy hunting but no human settlement. The LGCA allows human activities such as pastoralism, farming, and settlement. The MWR is a privately owned wildlife ranch which is bordered by NCA to the east, MGR to the north, and with SNP southern boundary being approximately 7 km north of the northern edge. The NCA allows multiple land use with seminomadic Maasai pastoralists practicing traditional livestock grazing while coexisting with wildlife (Veldhuis et al., 2019). For our analyses, we divided our study area in three major zones within the Serengeti ecosystem. Northern Serengeti (NSE) comprising of LGCA, IGR, GGR, Western and Central (SNP), Southern Serengeti (SSE) which consists of Southern SNP, MWR, MGR, and Ngorongoro Conservation Area (NCA).

#### 2.1.2 Tarangire‐Manyara ecosystem

The Tarangire‐Manyara ecosystem (TME) comprises three protected areas: Tarangire National Park (TNP), Lake Manyara National Park (LMNP), and Manyara Ranch (MANR) which acts a migratory corridor between the two parks (Figure 2). TNP and LMNP manage wildlife for tourism but, Manyara Ranch (MANR) is a private land conservancy managed for livestock grazing and wildlife tourism. Most of the land in the TME (~85%) fall under community land managed as open areas, game controlled areas, or wildlife management areas (Morrison, Link, Newmark, Foley, & Bolger, 2016), which allow different combinations of extractive and nonextractive wildlife uses, and human settlements and agriculture. A rift valley wall (500–1,000 m) at the western edge of the TME may prevent the free movement of wildlife to and from SE (Ahlering et al., 2012). The TME elephants were also affected by poaching before a ban on international trade in 1989. Since then, the population recovered to 4,202 individuals (TAWIRI, 2015) with an annual growth rate of 7% due to low poaching pressure and conducive climatic conditions (Foley & Faust, 2010).

#### 2.1.3 Ruaha and selous ecosystems

Ruaha ecosystem comprises of Ruaha National Park (RNP), Rungwa, Kisigo and Muhesi Game Reserves and Lunda‐Mkwambi Game Controlled Area. Ruaha National Park is the largest national park in Tanzania (Figure 2), and it covers 20,226 km^2^ (TANAPA, 2019). The Greater Ruaha River and Ihefu wetland are the primary source of water in the ecosystem. The RNP has diverse vegetation types from Miombo woodland that extends from Southern Africa to *Acacia* woodlands (TANAPA). The number of elephants in the Ruaha ecosystem in 2015 was 8,272 (TAWIRI, 2015). Selous Game Reserve (SGR) is the largest game reserve in Africa, located in the southeast of Tanzania at long. 38°15′E and lat. 7°35′S (Figure 2). Due to its size, SGR has eight administrative sectors. In this study, we focused on two sectors: Matambwe (MAT) and Kingupira (KPR) sectors. Matambwe is the only sector within the SGR that conducts photographic tourism. The rest of the SGR is designated for trophy hunting blocks. The Rufiji River separates Matambwe from Kingupira sector. The current number of elephants in the Selous ecosystem is 15,217 (TAWIRI, 2015). Between Ruaha and Selous ecosystems, there is Udzungwa Mountain National Park (Udzungwas), part of the Eastern Arc Mountains with a high level of species endemism and richness (Burgess et al., 2007; Dimitrov, Nogués‐Bravo, & Scharff, 2012). Between Udzungwas and SGR are the Kilombero Valley (6,650 km^2^) which is an important bird area and important farming areas especially for rice and sugar cane (Figure 2), and an area of conservation concern due to rapid habitat conversion.

### 2.2 Field data sample collection

We used DNA extracted from elephant fecal samples to genotype 11 microsatellite loci and mitochondrial DNA sequences of a hypervariable region in cytochrome b in the control region of D‐loop. To ensure a higher concentration of DNA, we only collected samples from fresh dung. Scrapings of 5g were taken from the outer layer of the dung bolus, where most of the epithelial cells are found, and stored in Queen's College Buffer in 50 ml conical tubes, following methods established by Ahlering et al. (2012). Where possible, 50 samples were collected from each site, but a minimum of 25 samples in small, isolated populations were obtained and the GPS location of each recorded. Samples were collected over a short time frame trying not to sample closely related individuals (Ahlering et al., 2012). We used COLONY (Jones & Wang, 2010) to identify resampled individuals that had identical multi‐locus genotypes. Samples were shipped to the Pennsylvania State University for genetic analyses. Each tube containing sample was sealed well using parafilm and placed into a zip‐locked bag to avoid cross‐contamination or leakage. Four tissue samples from the Tanzania Wildlife Research Institute (TAWIRI) for elephants that died naturally were used as a positive control for allele scores due to their higher DNA quality than that of fecal samples. All required permits were obtained from the United Republic of Tanzania and the US Fish and Wildlife Service.

### 2.3 DNA isolation

DNA was extracted and purified using company protocols for commercially available DNA extraction kits (QIAamp DNA Stool Mini Kit) with minor modifications (Eggert, Maldonado, & Fleischer, 2005). The initial volume of samples was increased from 200 µl to 1,000 µl and added 1,400 µl of buffer ASL and incubated at 55°C while shaking at 225 rpm for 12 hr (Lohay, 2019). Other steps followed QIAamp Stool mini kit protocol.

### 2.4 Microsatellite analysis

#### 2.4.1 PCR amplification and genotyping

We performed PCR amplification of microsatellite loci from fecal samples using QIAGEN reagents (QIAGEN multiplex PCR kit). Eleven loci that had previously been found to be polymorphic in elephants were used: LAT06, LAT08, LAT13, LAT24 (Archie, Moss, & Alberts, 2003), FH19, FH48, FH60, FH67 (Comstock, Wasser, & Ostrander, 2000), LA5, LA6 (Eggert, Ramakrishnan, Mundy, & Woodruff, 2000), and LafMS02 (Nyakaana, Okello, Muwanika, & Siegismund, 2005). Forward primers were labeled with one of three fluorescent dyes, FAM, NED, or VIC, (Appendix S1). Standard QIAGEN multiplex PCR protocol for PCR amplification was used. The availability of each primer pair to amplify a PCR fragment with predicted size range was validated via agarose gel electrophoresis. We estimated fragment lengths for each sample (allele) from digital gel images.

Having optimized our primer selection and amplification conditions, we grouped 11 microsatellite loci into four multiplexed panels for genotyping (Appendix S1). Initial microsatellite fragment detection of the multiplex PCR products was performed at the Genomic Core Facility of the Pennsylvania State University using ABI 3730xl DNA analyzer (Applied Biosystems). Individual microsatellite binning and scoring was conducted using GeneMapper^®^ v. 5 (Applied Biosystems), and individual alleles in each amplified loci were manually verified and compiled into working multi‐locus genotypes for each fecal sample collected. In order to ensure quality control standards, individual samples underwent two separate amplification, fragment analysis, and genotyping rounds, to avoid inconsistent allele patterns or weak peak fragment identification (Okello et al., 2005).

### 2.5 Genetic diversity and differentiation

To account for allelic dropout, null alleles, and scoring error due to stuttering, we used MICRO‐CHECKER 2.2.3 (Van Oosterhout, Hutchinson, Wills, & Shipley, 2004). Genotypes were then corrected based on MICRO‐CHECKER 2.2.3 results. Identical multi‐locus genotypes were identified using COLONY (Jones & Wang, 2010) and were removed from analysis as they were considered to be resampling of the same individuals. The number of alleles and their frequency was determined for all loci across individuals in all the populations using GenAIEx 6.502 (Peakall & Smouse, 2012). GenAIEX was also used to export files into formats compatible with other genetic analyses software. We also calculated population differentiation (F′ST; Bird, Karl, Smouse, & Toonen, 2011; Meirmans & Hedrick, 2011) and fixation indices (*F*_ST_) in GenAIEx version 6.502. We tested for deviations from expectations under Hardy–Weinberg equilibrium (HWE) and for linkage disequilibrium (LD) within protected areas in GENEPOP (Raymond & Rousset, 1995; Rousset, 2008). The Markov chain strategy was used with 1,000 dememorizations, 1,000 for combining independent test results across study location and the number of loci was used to determine the statistical significance test results. A Bonferroni correction for multiple comparisons was applied using a Holm‐Bonferroni sequential correction for both HWE and LD tests (Hochberg, 1988; Rice, 1989). Effective population size (Ne) from each location was estimated using the single‐simple linkage disequilibrium (LD) method in LDNe version 1.31 (Waples, Antao, & Luikart, 2014; Waples & Do, 2008). To estimate Ne, we used specific settings including a monogamous mating model (Coombs, 2010) under the LD method (Waples, 2006) and the jackknife method option was selected to obtain 95% confidence intervals (Weathers et al., 2019).

### 2.6 Population structure

The IBD program (Bohonak, 2002) was used to test whether there is evidence for isolation‐by‐distance (IBD). Individuals that are geographically close to each other tend to be more genetically related than distant individuals due to random mating. Spatial autocorrelation (*r*) was then plotted against geographic distance (km) to determine if the two variables were significantly correlated. FSTAT (Goudet, 1995) was used to calculate the inbreeding coefficient (*F*_IS_) and allelic richness (*A*_R_) which represents the number of alleles standardized to the smallest sample size in the study area. GenIEx (Peakall & Smouse, 2006, 2012) was used to calculate *F*_ST_ whereas ARLEQUIN (Excoffier, Laval, & Schneider, 2005) was used to assess genetic diversity by estimating the expected (*H*_E_) and observed (*H*_O_) heterozygosities. *F*_ST_ values were normalized by dividing pairwise *F*_ST_ to the geographic distance between subpopulations and denoted as *F*_ST_/km.

To determine elephant population structure in our samples, we used STRUCTURE 2.3 (Falush, Stephens, & Pritchard, 2003, 2007; Pritchard, Stephens, & Donnelly, 2000). This program uses a Bayesian clustering model to assign individuals to a population while simultaneously estimating population allele frequencies. This model aims to determine the number of subpopulations *K* within the population, where *K* in most cases is unknown (Pritchard et al., 2000). *K* was inferred by running ten iterations for each *K* value from 1 to 10 using an admixture model with a LOCPRIOR option. A burn‐in period at 1 × 10^6^ and Markov Chain Monte Carlo (MCMC) repetition value of 1 × 10^6^ was set. STRUCTURE HARVESTER was used to identify all primary (i.e., uppermost) Δ*K* estimates (Evanno, Regnaut, & Goudet, 2005) and subsequent nested population clusters using CLUMPAK (Kopelman, Mayzel, Jakobsson, Rosenberg, & Mayrose, 2015).

Following STRUCTURE, individual clusters were assessed for HWE conformance and *LD* significance since nonconformance in combination with significant levels of *LD* may indicate recent gene flow has occurred between clusters or that family groups were sampled. Spatial autocorrelation (*r*) implemented in GenAIEX (Peakall & Smouse, 2006, 2012) was conducted to test for the presence of spatial structure for the genetic and geographic datasets. Evenly spaced lag distance of 30 km was selected based on the sampling distribution and to provide a sufficient number of pairwise comparisons.

### 2.7 Mitochondrial sequencing and analysis

All 688 samples were sequenced to capture all existing haplotypes in the sampling locations but only 558 individuals were successfully sequenced (Table 1). 622 base pairs (bp) of cytochrome b gene were PCR amplified using forward primers MDL5 5′‐ TTACATGAATTGGCAGCCAACCAG‐ 3′ and reverse primers MDL3 5′‐ CCCACAATTAATGGGCCCGGAGCG‐ 3' (Fernando & Lande, 2000). The primers MDL5 and MDL3 amplify a 622 bp region of mitochondrial DNA including ~100 bp of cytochrome b, the transfer RNAs for threonine and proline, and ~350 bp of the control region or d‐loop. A different reverse primer, mtCR3 5′‐ GTC ATT AAT CCA TCG AGA TGT CTT ATT TAA GAG G‐ 3′, was used to samples that did not amplify successfully with the MDL3 reverse primer. PCR reactions were performed with the initial polymerase activation step at 95°C for 3 min, denaturation at 95°C for 30 s, annealing temperature at 60°C for 45 s, extension at 72°C for 30 s for 35 cycles. Each PCR mixture contained 3 μl of 5× Green GoTaq reaction buffer (Promega), a final concentration of 0.33 μM for each of the primers, 0.13 μM of dNTP (Quanta bio), Bovine serum albumin (BSA) 0.1 μg/μl, and 3 μl of DNA template of unknown concentration in a 15 μl volume. For each PCR reaction with test samples, we also ran a negative control (no DNA) and a positive control using DNA from known tissue samples. 6 μl of the PCR product was electrophoresed on at 2% agarose gel in Tris‐Acetate EDTA running buffer at 120V for 45 min and stained with GelRed (Biotium) so that the fragments could be visualized and photographed via a UV fluorescent gel documentation system. PCR products were sequenced using reverse primer only unless the sample did not have clean sequence results or had a unique haplotype. Sequence results in the trace file format were visually inspected using SnapGene^®^ software 4.2.4 (from GSL Biotech; available at snapgene.com) by comparing to a reference sequence for the hypervariable control region of elephants (Hauf, Waddell, Chalwatzis, Joger, & Zimmermann, 1999). All sequences were aligned using CLUSTALX2 (Larkin et al., 2007). Our sequences were compared with other published studies to identify unique haplotypes (Ahlering et al., 2012; Debruyne, 2005; Eggert, Rasner, & Woodruff, 2002; Ishida et al., 2013). Phylogenetic relationships among unique mtDNA were inferred using maximum parsimony (MP) analysis implemented in PAUP* 4.0b10 (Swofford, 2002). Support for the nodes for each analysis was assessed using 1,000 bootstrap iterations. We constructed a neighbor‐joining (NJ) tree from MEGA version 7 (Kumar, Nei, Dudley, & Tamura, 2008) to determine if both approaches produced tree with similar topology. Haplotype diversity (*h*) and nucleotide diversity (*π*) were calculated using Arlequin version 3.5 (Excoffier et al., 2005; Excoffier & Lischer, 2010) and constructed median‐joining (MJ) network PopArt 4.8.4 (Leigh & Bryant, 2015).

**TABLE 1:** Genetic diversity of African savanna elephants based on the sequence of 622 bp of mtDNA

Location	*N*	*H*	*h*	*π*
NSE	220	10	0.355	0.145
SSE	75	12	0.683	0.242
NCA	82	9	0.762	0.252
MAR	84	10	0.790	0.208
TNP	42	3	0.442	0.141
RNP	25	3	0.290	0.004
SGR	30	12	0.910	0.184

Note

The number of samples (*n*), number of haplotypes (*H*), haplotype diversity (*h*), and nucleotide diversity (*π*).

## 3 RESULTS

### 3.1 Microsatellite analyses

We successfully genotyped 714 samples of the 800 fecal samples collected between 2015 and 2017 (Appendix S4). We identified 26 samples with identical multi‐locus genotypes and were removed from the analysis. After removing the resampled data, 688 unique elephant samples remained (sampling locations and microsatellite genotypes: Dryad https://doi.org/10.5061/dryad.zs7h44j4m) and were used in the analyses of nuclear DNA. One locus, FH60, consistently showed evidence of a null allele for all sampling locations and deviated from HWE after applying a Bonferroni correction for multiple comparisons and was thusly removed from subsequent analyses, which therefore relied on 10 loci.

Genetic diversity indices are presented for seven zones and specific sampling locations within those zones (Table 2). Our population genetics results largely suggested that elephants sampled from the greater Tarangire‐Manyara ecosystem appear marginally, yet significantly isolated from elephants sampled across the more north‐western tier of Tanzania in the greater Serengeti ecosystem (mean *F*_ST_ = 0.03) (Tables 2 and 3). Despite observing evidence of significant isolation, the populations of elephants we examined generally exhibited robust levels with allelic richness (mean *A*_R_ = 9.96), heterozygosity (mean *µH*_E_ = 0.73) (Table 2), and effective population sizes (mean *N*_e_ = 148). Both NSE and SSE had the highest effective population size (*N*_e_) of 309 and 310, respectively, but the NCA and SGR had the lowest *N*_e_ of 95 elephants (Table 2). For RNP and SGR elephants, the lowest *F*_ST_ of 0.018 was observed between KPR and MAT whereas the highest *F*_ST_ was between KPR and RNP. The *F*_ST_ values between RNP and MAT were 0.26 which was lower compared with the *F*_ST_ value between KPR and RNP.

**TABLE 2:** Genetic diversity of African savanna elephants from 7 locations in Tanzania and 10 genotyped microsatellite loci

Protected area	*N*	*N*_a_	*A*_R_	*H*_o_	*uH*_e_	*F*	*F*_IS_	HWE	LD	*N*_e_ (CI)
Northern Serengeti Ecosystem (NSE)	212	11.0	9.78	0.72	0.73	−0.01	0.01	0.60	0.07	309 (223–464)
Southern Serengeti Ecosystem (SSE)	112	10.0	10.35	0.73	0.73	−0.03	−0.01	0.70	0.07	310 (312–524)
Ngorongoro Conservation Area (NCA)	104	10.2	10.23	0.70	0.72	0.00	0.02	0.40	0.20	95 (75–122)
Manyara Ranch Conservancy and Lake Manyara National Park (MAR)	98	10.2	10.47	0.71	0.74	0.03	0.05	0.60	0.29	114 (88–150)
Tarangire National Park (TNP)	64	8.5	9.22	0.71	0.73	0.01	0.03	0.70	0.02	111 (79–170)
Ruaha National Park (RNP)	48	8.5	9.29	0.70	0.74	0.03	0.05	0.70	0.02	114 (83–169)
Selous Game Reserve (SGR)	50	9.2	10.41	0.68	0.74	0.08	0.09	0.40	0.11	95 (64–161)
Mean	97.77	9.71	9.96	0.71	0.73	0.01	0.037	0.58	0.11	
Standard error	6.29	0.37	0.20	0.01	0.01	0.02	0.01	0.05	0.09	

Note

Number of samples (*N*), number of different alleles (*N*_a_), allelic richness (*A*_R_), observed heterozygosity (*H*_o_), unbiased heterozygosity (*uH*_e_), *F* = Fixation index, *F*_IS_ = inbreeding coefficient, proportion of loci conforming to Hardy–Weinberg equilibrium (HWE) and proportion of locus pairs in significant (*p* < .001) linkage disequilibrium (*LD*) and mixed cohort effective population size (*N*_e_) estimates are provided (CI = confidence interval).

**TABLE 3:** Pairwise genetic differentiation (*F*_ST_) for the African savanna elephants in Tanzania

Locations		NSE	SSE	NCA	MAR	TNP	RNP	SGR
NSE	*F*_ST_	–	0.111*	0.229*	0.388*	0.588*	0.569*	0.353*
	distance km		143	155	224	273	647	730
	*F*_ST_/km		7.76	14.77	17.32	21.54	8.79	4.84
SSE	*F*_ST_	0.007*	–	0.019*	0.126*	0.288*	0.362*	0.207*
	distance km	143		66	152	183	500	618
	*F*_ST_/km	0.51		2.88	8.29	15.74	7.24	3.35
NCA	*F*_ST_	0.009*	0.009*	–	0.056*	0.165*	0.235*	0.161*
	distance km	155	66		76	135	476	506
	*F*_ST_/km	0.61	1.31		7.37	12.22	4.94	3.18
MAR	*F*_ST_	0.021*	0.020*	0.013*	–	0.110*	0.153*	0.143*
	distance km	224	152	76		60	463	513
	*F*_ST_/km	0.96	1.34	1.75		18.33	3.3	2.79
TNP	*F*_ST_	0.040*	0.046*	0.028*	0.034*	–	0.031	0.339*
	distance km	273	183	135	60		429	449
	*F*_ST_/km	1.45	2.53	2.05	5.70		0.72	7.55
RNP	*F*_ST_	0.021*	0.021*	0.016*	0.019*	0.028*	–	0.384*
	distance km	647	500	476	463	429		333
	*F*_ST_/km	0.32	0.41	0.34	0.4	0.66		11.53
SGR	*F*_ST_	0.027*	0.029*	0.026*	0.032*	0.041*	0.029*	–
	distance km	730	618	506	513	449	333	
	*F*_ST_/km	0.37	0.46	0.52	0.62	0.92	0.87	
	mtDNA	Micr		RNP v NTmt	RNP v NTmicr	SGR v NTmt	SGR v NTmicr	
Mean *F*_ST_/km	8.78	1.09		4.99	0.43	4.34	0.58	

Note

The *F*_ST_ based on SSRs are below the diagonal and mtDNA are above the diagonal. Significant levels of *p* ≤ .05 are indicated with an asterisk. Normalized *F*_ST_ *t* was calculated by dividing *F*_ST_ to geographic distance (km), and the obtained ratios are in ×10^−4^. The mean *F*_ST_/km was computed to determine which normalized *F*_ST_/km deviated significantly from the mean.

Abbreviations: mt, mtDNA; Micr, microsatellites; NT, Northern Tanzania.

**FIGURE 3:**
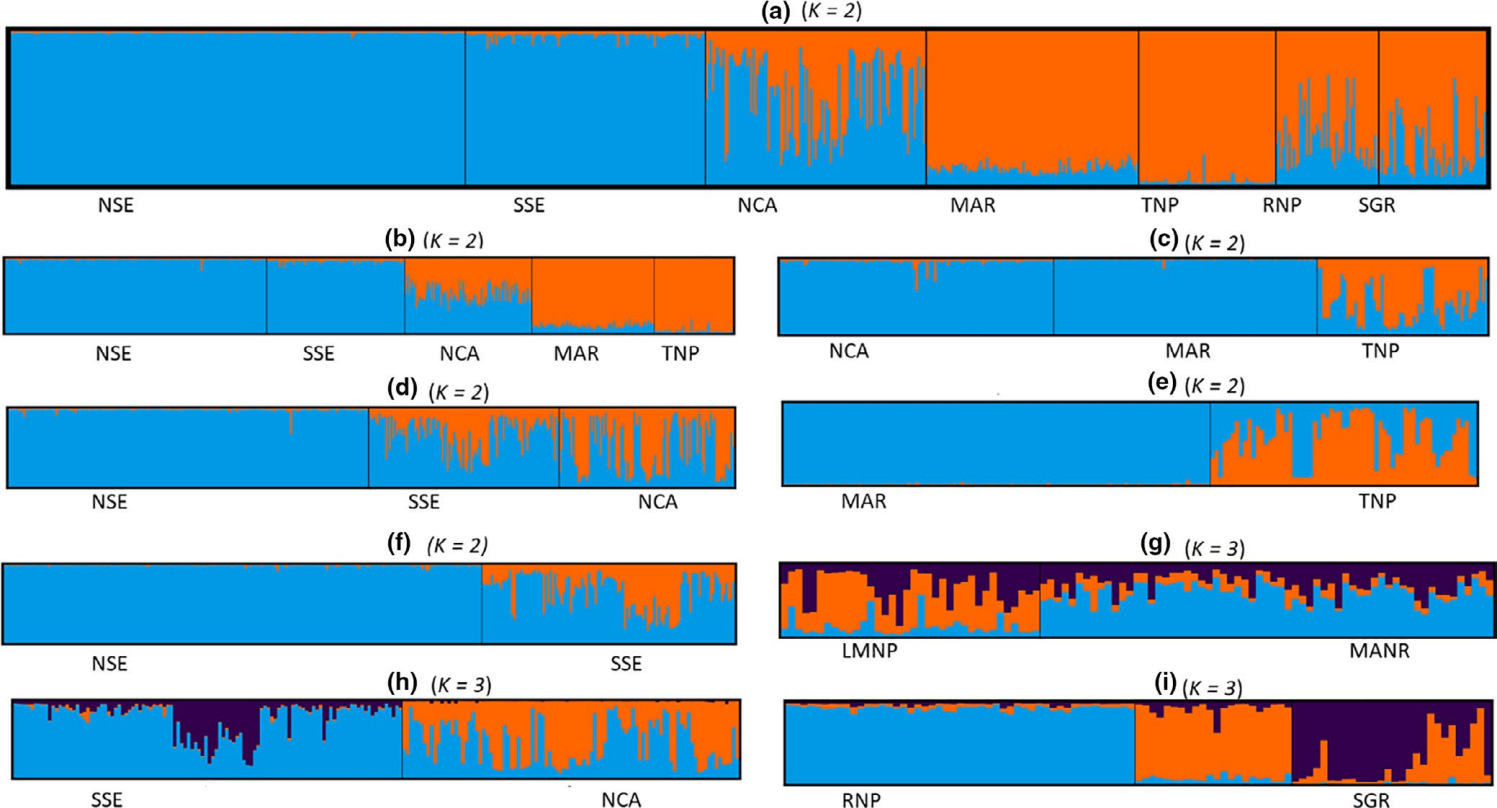
Hierarchical population STRUCTURE analysis for the African savanna elephants in Tanzania using 10 microsatellite loci. Each individual is represented by a thin vertical bar partitioned into color segments representing the individuals’ ancestry into subpopulations. The optimum number of clusters (*K*) was obtained using the Evanno method (Evanno et al., 2005). Estimated membership coefficient plots (a‐i) indicate the pattern of individual elephant cluster assignment for each of the hierarchical analysis. NSE, North Serengeti; SSE, South Serengeti; NCA, Ngorongoro Conservation Area; MAR, Lake Manyara National Park (LMNP) and Manyara Ranch (MANR); TNP, Tarangire National Park; RNP, Ruaha National Park, and SGR, Selous Game Reserve

Hierarchical STRUCTURE analysis indicated that the uppermost level of population structure was represented by two clusters (*K* = 2; Figure 3a; Appendix S2). The NSE and SSE formed one cluster (blue), and MAR, TNP, RNP, and SGR (orange) formed another cluster, as expected, the East African Rift separates these two clusters. NCA elephants, which are located immediately west and above the Rift wall, showed admixture between Tarangire and Serengeti ecosystems, which are on opposite sides of the Rift wall. Unexpectedly, further iterations of STRUCTURE analysis revealed two groups (Figure 3b,d,f) within the Serengeti Ecosystem: a northern subpopulation (NSE) and southern subpopulation (SSE). These subpopulations are relatively close, well within the range of elephant migration, and are absent of geographic or human imposed barriers. Three subpopulations were identified between the NCA and SSE which included Mwiba Ranch, Maswa Game Reserve, and Southern Serengeti National Park. However, there was weak support for genetic differentiation probably because there is gene flow between the NCA and SSE. Elephants in the Lake Manyara region (MAR) appear to be more closely related to those in the NCA across the East African Rift wall than they are to the neighboring Tarangire subpopulation (Figure 3c vs. e). Three subpopulations were identified within the MAR region but there was weak support of this clustering (Δ*K = *7; Figure 3g; Appendix S2). The SGR and RNP elephants formed three subpopulations (Figure 3i).

**FIGURE 4:**
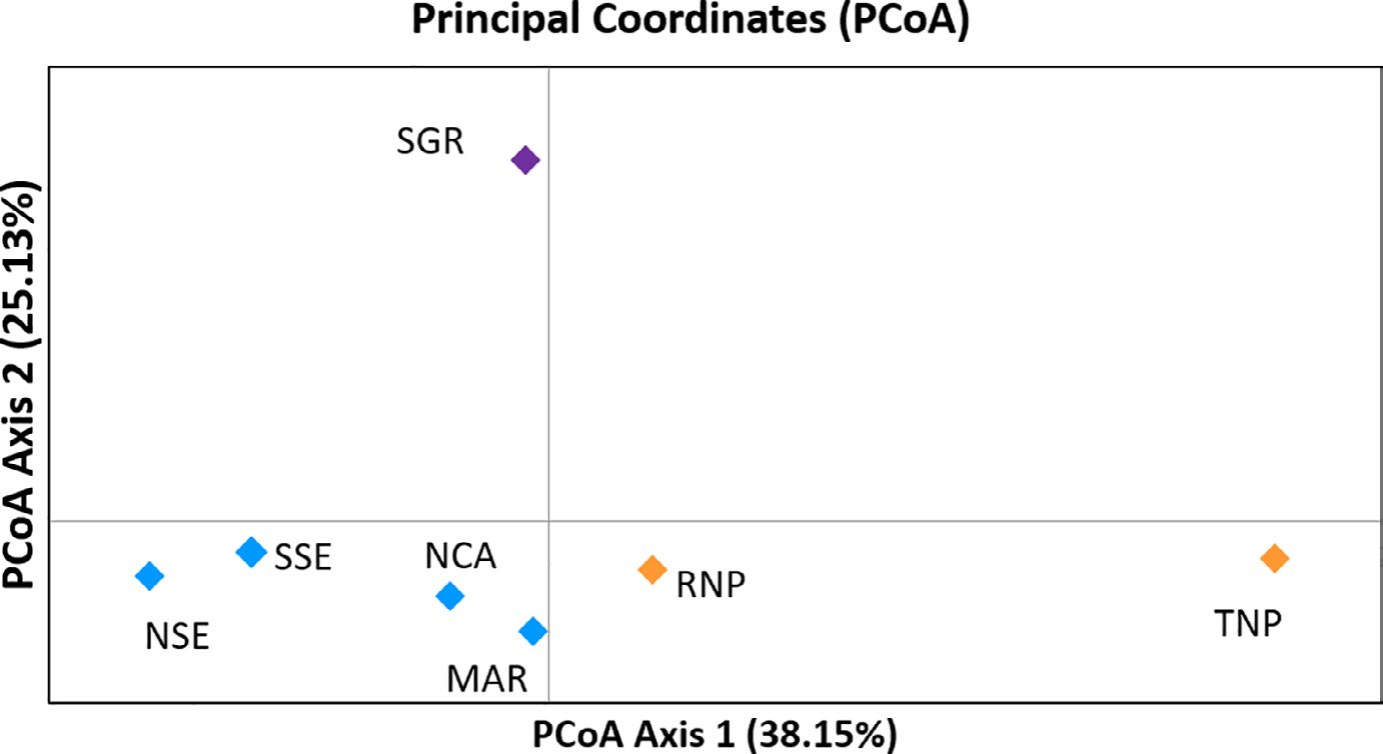
Principal coordinate analysis for 10 microsatellites using pairwise *F*_ST_ values in northern Tanzania for African savanna elephants. Colors used for ecosystems: blue = Serengeti, orange = Tarangire and Ruaha, and purple = Selous. Percentage of variation explained by axis 1, 2, 3 are 38.15, 25.13, and 15.83, respectively. The long form of the locations is shown in Table 1

Principal coordinate analysis (PCoA) was performed to determine the number of clusters using *F*_ST_ values and genetic distance matrix between all pairs of individuals. At least two clusters were identified in northern Tanzania as shown in Figure 4. Samples ordinated close to one another are more closely related than those ordinated far away. RNP and TNP elephants were clustered together while MAR was clustered with the SE. While the Nothern Tanzania sites (NSE, SSE, NCA, and MAR) and SGR clustered on axis 2, RNP was closer to them on axis 2 than to TNP. On Axis 2, SGR was seen as different than any other site (Figure 4).

**FIGURE 5:**
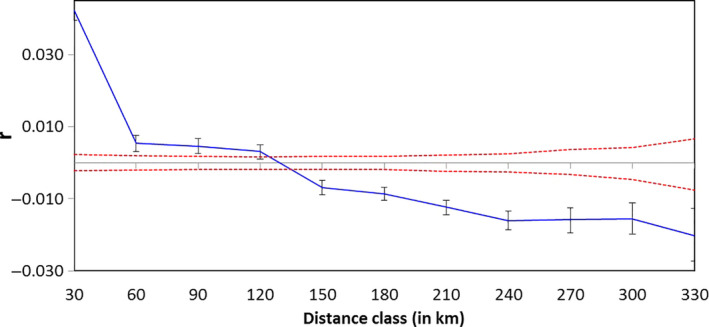
A correlogram showing spatial genetic autocorrelation (*r*) among elephants in northern Tanzania as a function of Euclidean distance. We defined distance classes every 30 km. Dotted lines indicate the 95% CI around the null hypothesis of no genetic structure. The error bars around r represent the 95% CI, as determined by bootstrapping (999 iterations)

Spatial autocorrelation (*r*) implemented in GenAIEX (Peakall & Smouse, 2012) was conducted to test for the presence of spatial structure for the genetic and geographic datasets. This analysis suggested the presence of spatial structure; the observed values of r were outside the upper and lower bound at a 95% confidence interval. A null hypothesis of no spatial structure for the genetic and geographic data sets was tested. Elephants within 120 km show genetic similarity with positive spatial autocorrelation (Figure 5), whereas beyond 120 km, elephants were less genetically similar (with negative *r*). Elephants that are closer to each other show higher genetic similarity than those far apart. Results from a Mantel test showed a positive correlation between genetic distance and geographic distance (*r* = .3719, *p* = .0008), as expected. However, one notable exception was the finding of a relatively high *F*_ST_ value between TNP and MAR subpopulations despite being only 60 km apart (Table 3). We found no significant correlation between geographic distance and genetic distance for mtDNA markers (*r* = −.1946, *p* = .3590) and nuclear loci (*r* = .9006, *p* = .1630) between Ruaha and Selous.

All *F*_ST_ values, except between RNP and TNP for the mtDNA, showed significant genetic differentiation (Table 3). The *F*_ST_ values from the mtDNA were higher than the nuclear DNA due to the differences in modes of inheritance. The average *F*_ST_/km for west versus east of the Rift wall for microsatellites was 9.568 and for mtDNA it was 1.042. The examination of specific pairwise *F*_ST_/km revealed several surprises including (1) the mtDNA *F*_ST_/km between TNP‐MAR was ~25× larger than the *F*_ST_/km between TNP‐RNP and the microsatellites *F*_ST_/km was ~9× larger. (2) Although the geographic distance between NCA‐MAR (76 km) is larger than the distance between MAR‐TNP (60 km) and the NCA and MAR are separated by the Rift wall, the microsatellites and mtDNA *F*_ST_/km values are approximately three times smaller. This strongly suggests that elephants have been traversing the Rift wall between the NCA and Manyara resulting in gene flow whereas gene flow between Manyara and Tarangire has been ablated.

### 3.2 Mitochondrial DNA analysis

Among the 688 unique samples, 558 successfully amplified the 622‐bp region of the mitochondrial DNA genome (Appendix S4). A total of 32 haplotypes (GenBank accession MN194226–MN194258) were identified of which seven had been published previously (Ahlering et al., 2012; Debruyne, 2005; Ishida et al., 2013), and 26 were unique to this study (Appendix S3). Haplotype diversity (*h*) ranged from 0.29 in RNP to 0.91 in SGR (Table 1). The nucleotide diversity ranged from 0.004 in RNP to 0.252 in NCA (Table 1). Overall nucleotide diversity was 2.63% which was similar to the continental‐wide level of 2.0% (Nyakaana, Arctander, & Siegismund, 2002).

**FIGURE 6:**
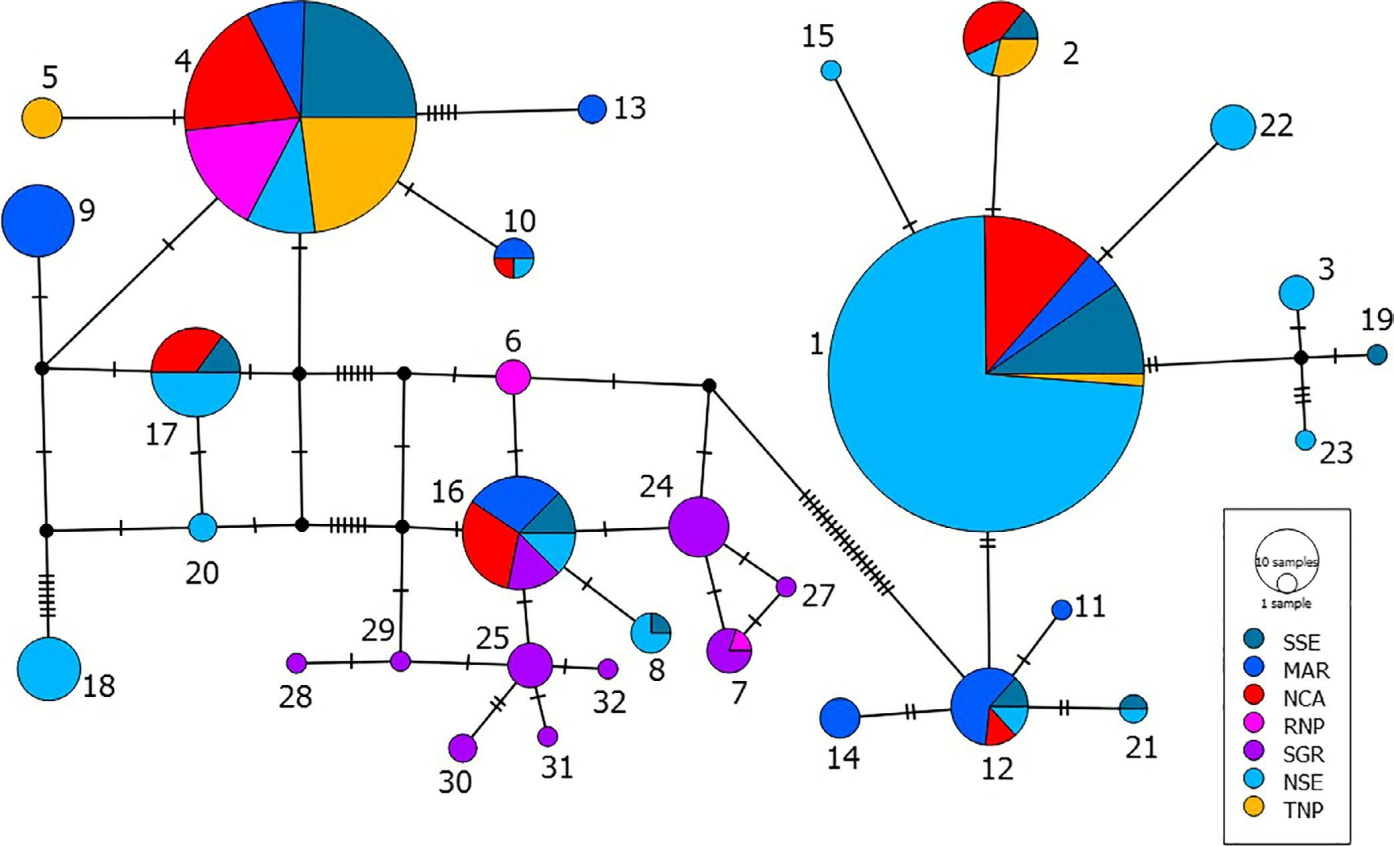
Median‐joining haplotype network based on mitochondrial DNA sequences from African savanna elephants in Tanzania. The size of circles is proportional to haplotype frequencies. The numbers 1–32 represent haplotypes identified in this study (Appendix S3). The stepwise mutations between haplotypes are indicated by hatch marks. SSE = South Serengeti, MAR = Manyara Ranch and Lake Manyara, NCA = Ngorongoro Conservation Area, RNP = Ruaha National Park, SGR = Selous Game Reserve, NSE = Northern Serengeti, and TNP = Tarangire National Park

**FIGURE 7:**
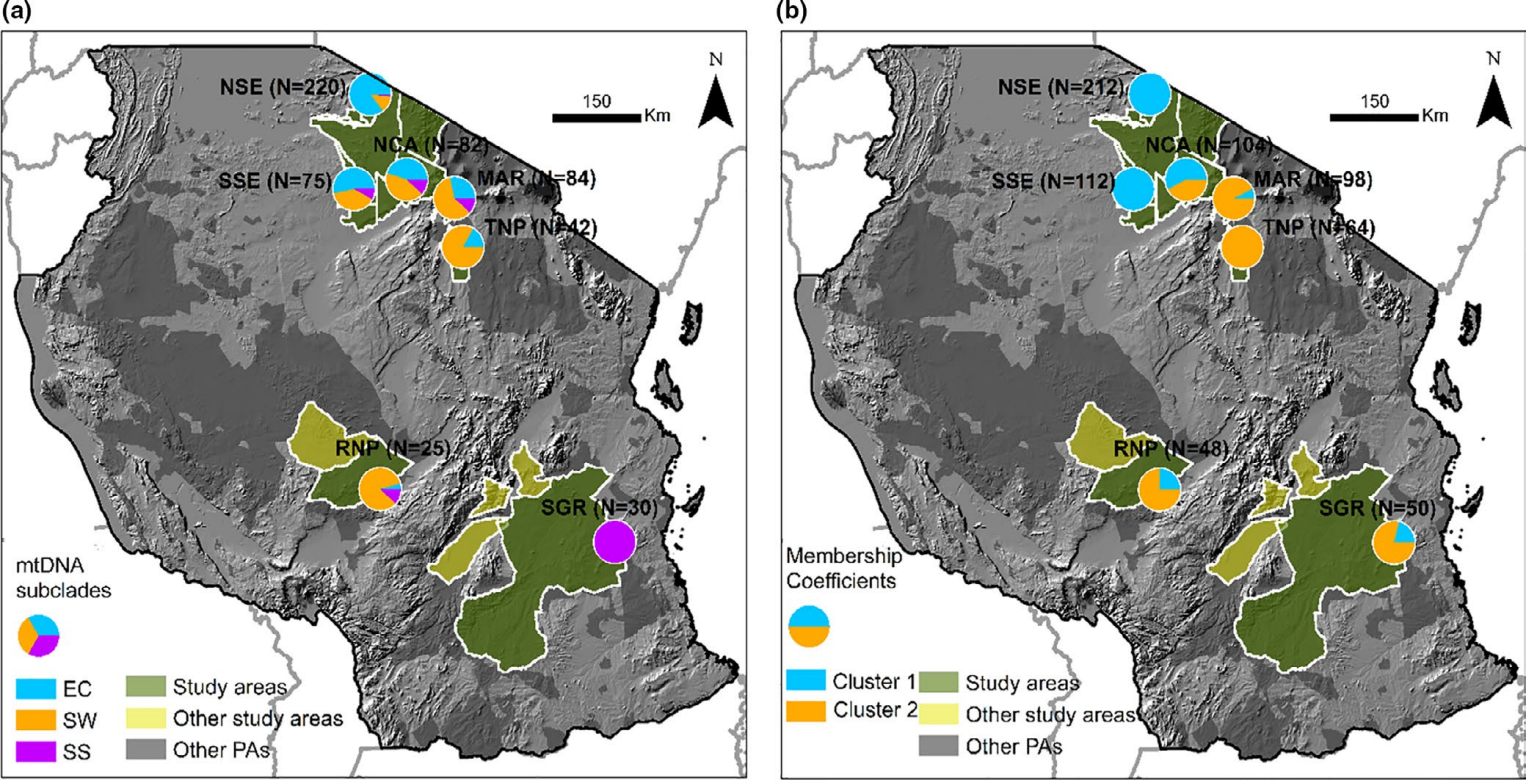
Map showing genetic relationships for African savanna elephants in Tanzania. (a) Membership coefficient (%) obtained from STRUCTURE program showing the proportion of individuals in Cluster 1 (Blue) and Cluster 2 (Orange). (b) The distribution of haplotypes grouped in the three subclades that comprise savanna elephants (EC = East‐central (blue), SW = Savanna‐wide (orange), and SS = Southeast‐savanna (purple)

All haplotypes fall into two clades, Forest (F), and Savanna (S), which is further subdivided into three subclades: Savanna‐wide (SW), East‐central (EC), and Southeast‐savanna (SS) (Ishida et al., 2013). We identified eight haplotypes in the Savanna‐wide subclade, 11 in East‐central, and 13 in the Southeast‐savanna. The F‐clade is highest in NSE compared with other parts east of the Rift, while the Savanna‐wide mainly was prevalent in the TME and RNP and southern Serengeti (SSE). Southeast‐savanna was dominant in the SGR (Figure 6.). A median‐joining network for the mtDNA haplotypes shows 22 mutations between the F‐clade and the S‐clade and seven variations between Savanna‐wide and the Southeast‐savanna which are both in S‐clade (Figure 6). The rift valley wall between SE and TME may explain the haplotype differences as observed in Figure 7. The Savanna‐wide subclade was less common in the SE and most common in the TME, while the East‐central haplotypes were common in the SE which is on the western side of the rift valley. However, we also observed remarkable differences in the haplotype distribution between MAR and TNP which are less than 60 km apart.

**FIGURE 8:**
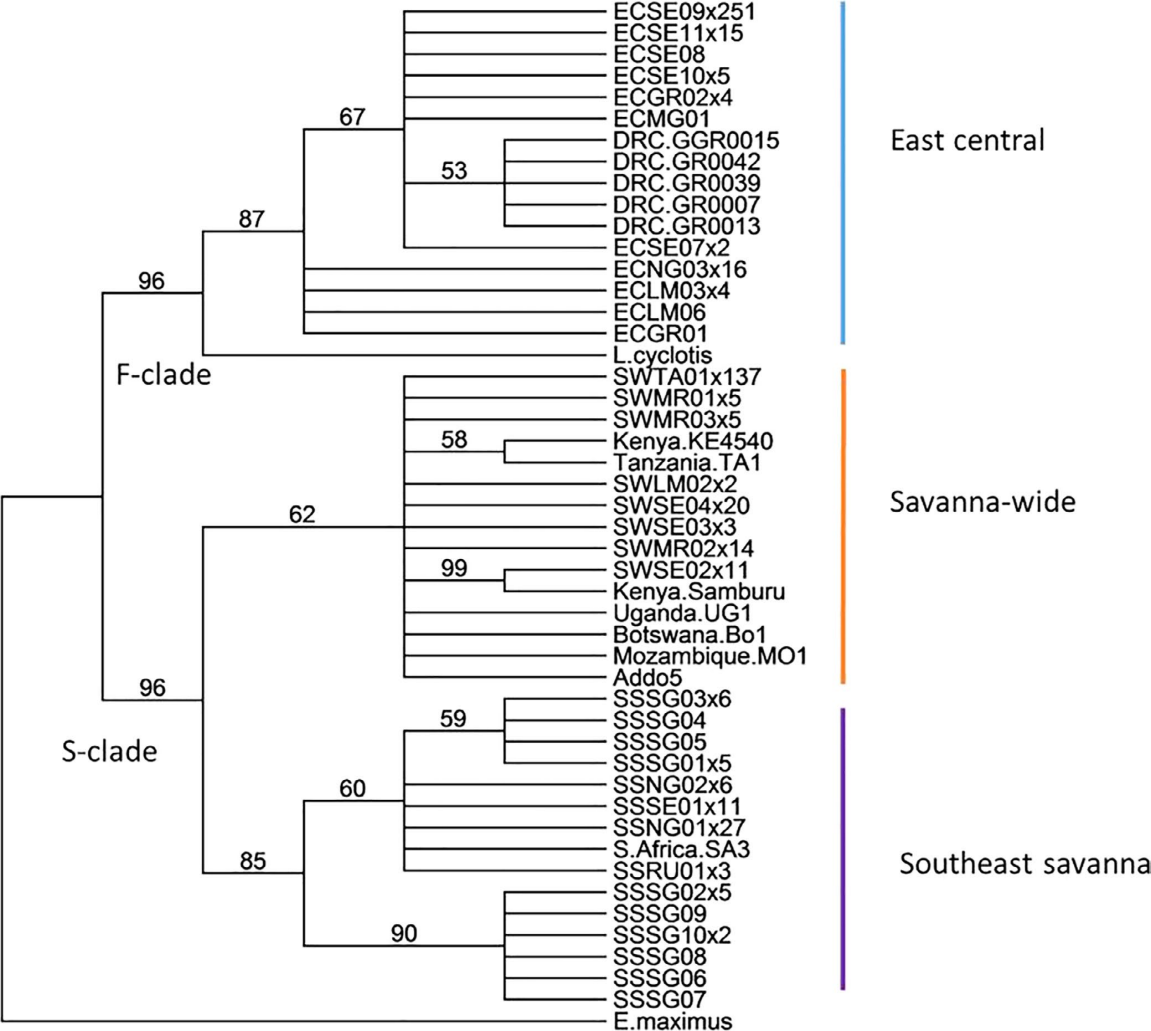
A neighbor‐joining tree constructed from 32 mitochondrial DNA sequences from this study and reference sequences from previous studies: GR0015‐0042, KE4540 (Ishida et al., 2013), UG1, BO1, TA1, MO1, and SA3 (Debruyne, 2005), Addo5 (Eggert et al., 2002), Samburu (Ahlering et al., 2012). Forest elephant (Eggert et al., 2002) and Asian elephant (Fernando et al. 2003) were used as outgroups. Blue = East‐central, Orange = Savanna‐wide, purple = Southeast‐savanna. Numbers on the branches represent the percentage bootstrap values > 50% for 1,000 replicates

Phylogenetic analyses of the 622‐bp mtDNA alignment using MP and NJ produced trees with similar topologies and we only presented MP (Figure 8). We detected a clear subdivision into F and S‐clades with 96% bootstrap values on both branches. The S‐clade was further subdivided into two subclades: savanna‐wide and southeast‐savanna (Figure 8). The savanna‐wide subclade only has 62% bootstrap and our support for this subclade is not as strong as the other two. Our data also suggest a clear pattern of haplotype distribution between northern and southern Tanzania. One hundred percent of Selous samples carried haplotypes with the southeast‐savanna (Figure 8), whereas the majority of elephants in the SE carry haplotypes in the F‐clades which is shared with the forest elephants (Ishida et al., 2013) and was not observed in Ruaha and Selous. Ruaha elephants had only three haplotypes (one unique) whereas in Selous GR there were ten haplotypes (eight unique) (Appendix S3). In Ruaha, one haplotype was carried by 84% of individuals sampled, and only one haplotype was unique to Ruaha. Only one was shared between Ruaha and Selous. In Selous, eight out of ten haplotypes were unique to Selous, of which two were previously published (Debruyne, 2005; Ishida et al., 2013).

## 4 DISCUSSION

Tanzania is bisected by the East African Rift, with major elephant populations west of the Rift in the greater Serengeti ecosystem and populations east of the Rift including Manyara, Tarangire, and Ruaha National Parks and the Selous Game Reserve and adjoining protected areas. A hierarchical STRUCTURE analysis of our genetic data indeed identified two metapopulations divided by the Rift. When the analysis is restricted to northern Tanzania, excluding Ruaha and Selous, the result is the same: The first level of STRUCTURE analysis identifies two metapopulations. Both nuclear and mtDNA results revealed that the Tarangire‐Manyara Ecosystem (TME) and the Serengeti Ecosystem (SE) are genetically distinct with limited gene flow among them. All pairwise *F*_ST_ values for microsatellites and all mtDNA showed significant genetic differentiation. mtDNA haplotype distribution was significantly different between SE and TME. The East African Rift acting as a geographic barrier between TME and SE accounts for at least some of the distributions of mtDNA haplotypes and microsatellites genotypes. However, habitat fragmentation and habitat loss between these two ecosystems are likely to have accelerated this genetic differentiation. While these data suggest that the Rift impedes gene flow, a closer inspection of the genetic differentiation of subpopulations adjacent or near the Rift revealed a more complex story. Elephants east and immediately adjacent to the Rift in the Lake Manyara region exhibit only one‐third as much differentiation with elephants in the Ngorongoro Conservation Area than they do with elephants in Tarangire, even though Lake Manyara and Tarangire are geographically closer and both east of the Rift (developed later in the discussion).

In contrast, we found that geographic normalized genetic distance (*F*_ST_/km) was twenty‐five times lower between Tarangire and Ruaha elephants than for Tarangire and Manyara. This suggests extensive gene flow over the long distance (>400km) between Tarangire and Ruaha prior to the eventual closure of corridors between them that occurred over the past 30 years (Riggio & Caro, 2017; Caro, Jones, & Davenport, 2009). It takes multiple generations for the effects of genetic differentiation to reflect in populations that were once connected. For this reason, we still see low or no genetic differentiation between Ruaha and Tarangire. The discrepancy between *F*_ST_ values based on mtDNA versus microsatellites is notable. The “mtDNA loci are generally a more sensitive indicator of population structure than are nuclear loci, and mtDNA estimates of *F*_ST_‐like statistics are generally expected to exceed nuclear loci” (Zink & Barrowclough, 2008). Differences in the mutation rates of nuclear versus mtDNA markers may explain this discrepancy. Nuclear mutation rates are generally lower than mtDNA mutation rates (~ a factor of 10; Brown, 1983; Zink & Barrowclough, 2008), and *F*_ST_ estimates are products of events occurring over a period four times longer than for mtDNA and effective population (*N*_e_) size for nuclear loci will frequently be four times that of mtDNA loci (Zink & Barrowclough, 2008). However, nuclear markers such as microsatellites have more resolving power than does mtDNA (Edwards & Bensch, 2009).

West of the Rift in the greater Serengeti ecosystem (SE), our microsatellite analysis provides insights into the presence of genetic differentiation within the Serengeti. Elephants from northern Serengeti (NSE) formed one cluster whereas elephants from southern Serengeti (SSE) formed another cluster. Pairwise *F*_ST_ values between the SSE and NSE were significant, suggesting genetic differentiation within the Serengeti ecosystem, probably resulting from recolonization by two different source populations in the 1950s, following local extirpation in the early part of the century (Sinclair, 2012).

Analysis of the hypervariable control region in D‐loop of the cytochrome b gene of mtDNA has frequently been used to assess nucleotide and haplotype diversity among mammals. The mtDNA is inherited in a haploid fashion because it is passed through the maternal lineage. Majority of Serengeti elephants carry haplotypes in the F‐clade which is also found among forest elephants. The presence of F‐clade in Serengeti indicates many generations of hybridization between forest and savanna elephants during the geological history of Africa (Ishida et al., 2011). “The historical reproductive success of female hybrids can be inferred by the presence of F‐clade mtDNA, derived from forest elephants in savanna herds” (Ishida et al., 2011). The Serengeti has two distinct populations that arrived from outside the Serengeti in the early 1960s as part of the population expansion that began in the 1950s after 100 years of near extermination due to the ivory trade from 1840 to 1890 (Sinclair, Hopcraft, Olff, & Mduma, 2008). Our results support this hypothesis because in the Serengeti we observed two main subclades, savanna‐wide, and east‐central. The savanna‐wide subclade dominates the eastern rift valley, whereas the east‐central subclade is predominantly found on the western side of the rift. Because mtDNA is maternally inherited, the observed pattern reflects females’ complex social structure.

Interestingly, Ruaha elephants which are more than 400 km away from Tarangire, share the same haplotypes with Tarangire elephants, and they are distinct from Selous GR, which is much closer geographically. This suggests historical female‐mediated gene flow between Ruaha and Tarangire, and the similarity of nuclear loci supports both high gene flow by both sexes. Also, there is evidence for gene flow between elephants in Serengeti ecosystem with Ruaha. While the Northern Tanzania sites and SGR clustered on axis 2, RNP was closer to them on axis 2 than to TNP. On Axis 2, SGR was seen as different than any other site (Figure 4). Probably elephants previously moved from southern Serengeti to Ruaha without passing through Tarangire. Unfortunately, wildlife corridors between RNP and TNP and between RNP and Serengeti are now entirely blocked (Riggio & Caro, 2017).

### 4.1 Tarangire‐Manyara corridor

Within the Tarangire‐Manyara ecosystem, we detected significant genetic differentiation: Manyara formed one cluster, and the Tarangire elephants formed a second cluster. Both nuclear and mitochondrial DNA data showed significant genetic differentiation. Our results suggest limited gene flow between Tarangire and Manyara probably due to increased habitat loss along wildlife corridors that previously connected these ecosystems, and which have been known to be under extreme threat for decades (Mwalyosi, 1991). However, Manyara elephants showed a higher haplotype diversity than Tarangire probably because there was gene flow between Manyara and Ngorongoro. In addition, Tarangire had only three haplotypes while Manyara had ten haplotypes. In Tarangire, one haplotype was carried by 35 individuals (83.33%) of all samples and only two haplotypes were shared between Tarangire and Manyara. This is also unexpected because the Manyara population is relatively low compared with Tarangire. Perhaps the high diversity in Manyara is due to both gene flow with the NCA and a much higher population size in the 1970s (Douglas‐Hamilton, 1973). One would predict that this diversity will decline significantly over the next few generations as a consequence of inbreeding due to loss of corridors to the NCA (O'Brien et al., 1985).

Furthermore, when we compared haplotype distributions between the east and west of the Rift wall, we found that most Manyara elephants shared haplotypes with Ngorongoro elephants. We detected a significant difference in haplotype distribution. For example, in Tarangire, only four elephants were carrying a haplotype in the East‐central subclade, but these haplotypes were common in Manyara. In Tarangire, there were no elephants carrying haplotypes in the southeast‐savanna subclade, but haplotypes in the East‐central subclade were observed in Manyara (Figure 6). It appears there is limited female‐mediated gene flow between Manyara and Tarangire although there is no landscape barrier between them, other than an increasing human footprint. Alternatively, there could be cultural or behaviorally mediated barriers to genetic connectivity among subpopulations. Females’ social behavior of remaining in their natal groups might drive discontinuities among subpopulations. The only viable migratory route between Tarangire and Manyara Ranch based on GPS data is the Kwa Kuchinja corridor (Kikoti, 2009). Kwa Kuchinja corridor has experienced a substantial increase in cultivation over the past decade and remains under heavy development pressure from agriculture and settlements (Morrison et al., 2016) thereby reducing connectivity between the Tarangire and Manyara Ranch populations.

### 4.2 Ngorongoro‐Manyara corridor

Wildlife corridors between the Tarangire‐Manyara and Serengeti ecosystems are under critical risk of being blocked completely (Lobora, Mduma, Foley, & Jones, 2010). The corridors between Lake Manyara National Park (LMNP) and the Ngorongoro Conservation Area (NCA) are essential for connecting the two ecosystems. Effects of habitat fragmentation on wildlife corridors between NCA and LMNP can be traced back to the 1940s when tsetse eradication allowed the expansion of human settlement in the Mbulu areas blocking the forest corridors (Homewood & Rodgers, 1991). Concerns about loss of corridors have been ongoing in the ecosystem since the 1970s. Increased human activities, especially agricultural settlement around Lake Manyara (Borner, 1985; Douglas‐Hamilton, 1973), threaten the viability of wildlife corridors and the expansion of Mto wa Mbu agricultural settlement is of particular concern (Mwalyosi, 1991). Wildebeests in Tarangire‐Manyara are distinct from the Serengeti without mixing for thousands of years, separated by the rift, whereas wildebeest have been migrating between Tarangire‐Manyara and Lake Natron to the North (Morrison & Bolger, 2014; Morrison et al., 2016).

The Ngorongoro elephants show admixture with elephants from Lake Manyara NP, which is also geographically very close. This admixture indicates recent gene flow between these subpopulations. However, most corridors have likely been lost due to intense agriculture on top of the rift (Table 4). Our study provides some insights into historical and contemporary gene flow between the two protected areas. There are limited number of natural corridors across the rift but these natural corridors are also compatible with agriculture. Consequently, human settlements have rapidly expanded into these limited natural corridors and blocked seasonal migration of elephants between the Ngorongoro/Serengeti and Lake Manyara. Although the Rift wall may impede the movement of elephants, there is enough evidence that there was gene flow between the two protected areas, including recent telemetry data from at least one male which moved over the rift from Natron to Loliondo GCA (Kikoti, 2009). In contrast, it appears that there was little mixing between Tarangire and Manyara. Now that the corridors between the NCA and Manyara are largely blocked, Lake Manyara elephants are likely completely isolated. The Lake Manyara population is small and therefore at greater risk of extinction through inbreeding depression and population decline through demographic stochasticity (Gilpin & Soulé, 1986). Historically, the Manyara National Park had the highest known elephant density in Africa (Douglas‐Hamilton, 1973). Elephants declined from ~500 in 1984 to about 150 in 1988 and then to 36 in 2007 and 34 in 2014 (Blanc et al., 2007; Kioko, Zink, Sawdy, & Kiffner, 2013; Prins, Der Jeugd, & Beekman, 1994; TAWIRI, 2015). Douglas‐Hamilton (1973) reported that the Manyara elephant population was young and fertile and was expanding at the rate of 3%–4% annually during the 1960–70s. However, the population size of elephants has remained around the 30 since 2007. Most African elephant populations were affected by poaching in 1980s but some protected areas such as Tarangire have recovered from poaching. Although the Lake Manyara population is small and likely isolated now, genetically it appears to be fairly robust. Heterozygosity values and allelic richness are similar to other populations, and the inbreeding coefficient is low (Table 2). Because elephants have long generation times, it will take time for the loss of genetic diveristy to manifest as inbreeding depression. Inbreeding depression and isolation may negatively impact the Lake Manyara elephants in future because the park is too small to maintain viable population sizes without connectivity to surrounding areas.

**TABLE 4:** A summary of main results relevant to stakeholders to show where the focus will be required to restore or protect wildlife corridors in Tanzania (Caro et al., 2009; Mduma et al., 2010)

Protected areas	Main results	Conservation actions/recommendations
Serengeti NP vs. Grumeti GR and Loliondo GCA	High genetic similarity between these protected areas	There is enough evidence of gene flow between these areas. No major conservation issues identified (Kikoti, 2009)
Ngorongoro CA vs. Maswa GR and Mwiba Ranch (SSE)	Three clusters identified but there was weak support of genetic differentiation There were a lot of shared mtDNA haplotypes	Areas south‐west of the Ngorongoro particularly Endulen, Esere, Kakesio are essential for connecting elephants with Mwiba Ranch and Maswa GR. The Ngorongoro Conservation Area Authority should increase protection in these areas and reduce human settlement in some areas that are frequently used by elephants.
Ngorongoro CA and Manyara NP	Both mtDNA and microsatellite data show high genetic similarity between Ngorongoro and Manyara	A corridor between Lake Manyara to Ngorongoro through Karatu is completely blocked. The best option is to use the corridor south of Lake Manyara NP through Marang Forest to South of Ngorongoro. This corridor was gazettted but human encroachment has occurred in these areas (Kisui, Bernard per.comm). There is evidence that the Silela corridor (north of LMNP) is still open, and there is likely movement along the escarpment by Eyasi. Elephants can still move up through Marang Forest, but they are then blocked by agriculture in the highlands before they can reach the Northern Highland Forest Reserve. Silela corridor is also open and being used but under threat (Morrion& Bolger 2014; Morrison et al., 2016)
Lake Manyara and Manyara Ranch	Weak support for genetic differentiation	Genetic evidence suggests recent or ongoing gene flow between these two areas. However, increasing number of settlements and expansion of Mto wa Mbu town between Manyara Ranch and Lake Manyara could pose a significant threat to this corridor. The government should discourage farming around these areas and keep the traditional pastoralism practiced by Maasai people for many years or include these villages in a WMA.
Manyara and Tarangire	Limited gene flow between these protected areas Only two haplotyepes were shared between Manyara and Tarangire. Manyara had significantly higher haplotype diversity than Tarangire	Historically there were at least 9 identified corridors (Mwalysosi, 1991). Now there are about three wildlife corridors connecting these protected areas: Kwakuchinja Corridor through Burunge WMA, Mswakini Chini and Mswakini Juu. Apart from Kwakuchinja which is currently secured after establishing a WMA, other wildlife corridors are threatened. There is evidence for movement of elephants between Tarangire and Manyara Ranch but our data suggest limited gene flow. Immediate action needs to be taken now to protect these wildlife corridors. These corridors are essential to connect Tarangire and Serengeti ecosystems.
Tarangire and Ruaha	Both microsatellite loci and mtDNA show genetic connectivity between Tarangire and Ruaha	Wildlife corridors between these protected areas seem to be blocked completely (Riggio & Caro, 2017). More research needs to be done to determine if there are any remaining movements of elephants between these ecosystems
Ruaha and Selous	Only one haplotype was shared between Ruaha and Selous. Three clusters were identified between Ruaha and Selous. The Eastern Arc Mountains seem to act as a barrier between these two ecosystems	A more intensive genetic study needs to be done between Ruaha and Selous. We support recommendations provided by Jones et al. (2012) to restore corridors between the two ecosystems, particularly between Selous and Udzungwa through Kilombero reserve

### 4.3 Connectivity between Ruaha and Selous

Our genetic data show that elephants from Ruaha and Selous are divided into two divergent mtDNA lineages: savanna‐wide and southeast‐savanna subclades. Similarly, nuclear loci data show significant population differentiation between Ruaha and Selous. Even within the Selous GR, between Matambwe and Kingupira sectors, two subpopulations were detected, implying limited gene flow within the reserve. A small proportion of admixture was observed in Matambwe sector from the STRUCTURE analysis, and the pairwise *F*_ST_ values were lower between Matambwe and Ruaha than Kingupira and Ruaha using microsatellites, indicating recent gene flow between them.

Male‐mediated dispersal has been documented among elephant populations in both Uganda (Nyakaana & Arctander, 1999) and Kenya (Okello et al., 2008). In both studies, there was a lack of congruence between mitochondrial DNA and nuclear‐based variation. Mitochondrial DNA data showed significant differentiation, whereas nuclear data showed low genetic subdivision between populations (Nyakaana & Arctander, 1999; Okello et al., 2008). This difference between markers was attributed to differences in the modes of mutations between mitochondrial and nuclear markers. Female elephants stay in family groups while males are ejected from groups after sexual maturity. The home range for elephant family groups is between 15 and 52 km^2^ (Douglas‐Hamilton, 1973). Thus, mitochondrial markers would likely remain restricted to specific localities (Nyakaana & Arctander, 1999). Although this difference in haplotype frequency was expected between Ruaha and Selous, lack of shared haplotypes could be attributed to the presence of the Eastern Arc Mountains, separating the two ecosystems. Furthermore, there was no significant correlation between genetic and geographic distance.

Genetic differences between Ruaha and Selous have likewise been noted in other species. A recent study on lions (*Panthera leo*) in Tanzania uncovered significant genetic differentiation between lions of Selous and Ruaha (Smitz et al., 2018). This differentiation could be a combined effect of both anthropogenic and environmental/climatic factors (Smitz et al., 2018). The presence of the Eastern Arc Mountain chains associated with the land use patterns may present a significant biogeographical barrier to lion dispersal (Smitz et al., 2018). Similarly, phylogenetic analyses based on mitochondrial DNA of sable antelope *(Hipotragus niger*) identified unexpected clear, distinct lineages between Ruaha and Selous which was attributed to the presence of the Eastern Arc Mountains (Pitra, Hansen, Lieckfeldt, & Arctander, 2002). Furthermore, Pitra et al. (2002) found that the initial allopatric fragmentation is geographically consistent with the discontinuous distribution of miombo habitats inside and outside of this montane circle in East Africa. There is also a clear difference in the vegetation cover between the western (Ruaha) and eastern (Selous) side of the Eastern Arc mountains. The Ruaha ecosystem is dominated by *Acacia* savanna vegetation while the Selous ecosystem is dominated by miombo woodlands. Our study also showed a similar pattern of elephant divergence, particularly from mitochondrial DNA haplotypes. All elephants from Selous were in a different mtDNA subclade.

A similar pattern was observed in the distribution of mitochondrial DNA haplotypes in northern Tanzania which were separated by the rift valley (Ahlering et al., 2012; Ishida et al., 2013). Elephants west of the rift carry different haplotypes from the east. However, in northern Tanzania, there were more shared haplotypes between the western and eastern side of the rift valley than between Ruaha and Selous that are separated by the Eastern Arc Mountains. Elephants can climb relatively steep slopes despite the rigidity of their joints (Lindsay & Lee, 2006). However, elephants are also known to avoid areas with steep slopes (Wall, Douglas‐Hamilton, & Vollrath, 2006). For that reason, mountain ranges may act as barriers for elephants in some cases (Epps et al., 2013; Wall et al., 2006). Elephants can negotiate relatively steep slope over the short distances, but long‐distance movement over steep terrain may be restricted by energetic limitations (Wall et al., 2006). These barriers are semipermeable to the movement of elephants (Sawyer et al., 2013).

Female elephants are philopatric and remain with their natal herd (Archie et al., 2007) whereas males are ejected from the herd upon sexual maturity and subsequently facilitate gene flow between herds (Archie et al., 2007). Using nuclear markers, there was significant genetic differentiation but low *F*_ST_ between Matambwe in the Selous and Ruaha, suggesting some amount of gene flow. Nuclear genes are not subject to the same inheritance limitations as mitochondrial DNA because males disperse nuclear markers (Brandt et al., 2014). Our data indicate male‐biased gene flow, with little evidence for female‐mediated gene flow between Ruaha and Selous. One of the haplotypes that Selous shares with other sites (SSNG01) is relatively common, being found in 5 other sites, while the second one (SSNG02) is found in 2 other sites and is more common in NCA than in Selous. While one explanation for this may be unidirectional colonization of elephants from Selous to Ruaha to northern Tanzania, another might be the retention of formerly widespread haplotypes prior to broad‐scale habitat fragmentation. No elephants in Selous carried haplotypes that were present in Ruaha and northern Tanzania.

### 4.4 History of elephant recolonization in Northern Tanzania

Mitochondrial DNA has been used to infer the ancestry of populations because it is inherited in haploid fashion. There is a hypothesis that elephants which recolonized the Serengeti came from two sources, one from the south and the other one from the north (Sinclair et al., 2008) based on anecdotal observations. Our mitochondrial DNA results demonstrate clear genetic differences in elephants in northern and southern Serengeti, which support the hypothesis of different elephant groups recolonizing the Serengeti from north and south. Our results affirm that there are at least two source populations that colonized the Serengeti: elephants with F‐clade haplotypes and those with S‐clade. Most elephants in the Serengeti carry haplotypes in F‐clade whereas most elephants in the Tarangire‐Manyara, Selous, and Ruaha carry haplotypes that fall in S‐clade. Most elephants from the Serengeti carry similar haplotypes with Bili Forest elephants in the Democratic Republic of Congo and Garamba elephants located in the Guinea‐Congolian/ Sudanian area (Ishida et al., 2013). Interestingly, these genetic differences inherited from the founder populations in the 1950s are still evident in the genetic structure of Serengeti elephants today, despite there being no geographic barriers that separate the northern and southern Serengeti populations.

### 4.5 Implications for conservation

The full consequences of habitat fragmentation on population structure for species with long‐life spans and generations may take time to be observed, because there is a time lag between changes to habitat and when the full implications of these changes are experienced by wildlife (Bennett, 1998, 2003). However, Lobora et al. (2018) identified early signs of genetic differentiation among young cohorts of elephants in south‐western Tanzania due to habitat fragmentation of miombo woodland. In recent years, the analysis of the structural connectivity of protected areas has been done at the national level (Riggio & Caro, 2017). At the policy level, the Tanzanian government passed wildlife legislation aimed at protecting migratory wildlife corridors in 2008 (Tanzania, 2008). Our results provide evidence of where Tanzania government needs to act to restore recently lost or protect still viable corridors for elephants (Table 4). In particular, we showed high genetic similarity between Ngorongoro and Manyara elephants, indicating at least previous connectivity between these populations. However, the corridors that facilitated this gene flow have been under threat from human habitat modification for decades, to the extent that it is no longer clear whether any remain open. We strongly recommend immediate action to restore wildlife corridors between Ngorongoro and Lake Manyara and between Tarangire and Manyara Ranch. Various studies have documented threats facing wildlife corridors in Tanzania (e.g., Caro et al., 2009; Lee, Bond, Kissui, Kiwango, & Bolger, 2016; Morrison & Bolger, 2014; Newmark, 2008; Riggio & Caro, 2017). However, minimal mitigation efforts have yet to take place. Likewise, our study has shown high genetic similarity between Ruaha and Tarangire elephants, but the corridors between them are now completely blocked (Table 4). The possible detrimental effects of this recent isolation on these two previously intermingle populations will take many years to become manifest due to the slow rate of genetic variation decay and the animals’ long‐life span. Thus, we cannot assume that observed low genetic differentiation between the Ruaha and Tarangire elephants is a reliable indicator of the present state of corridor activity because the genetic similarities reflect the past and not the present state of gene flow between these populations of elephants. While restoration of these corridors may be challenging, efforts to increase connectivity between Tarangire and Swagaswaga Game Reserve (found between Tarangire and Ruaha) could increase dispersal areas for elephants.

Maintaining genetic connectivity between protected areas is crucial for long‐term survival of species (Luikart, Ryman, Tallmon, Schwartz, & Allendorf, 2010). Unfortunately, the corridors connecting the Ruaha and Selous ecosystems are entirely blocked. Strategies for restoration are needed (Jones et al., 2012). However, female elephants may have been isolated long before the current habitat fragmentation caused by humans. Lions and sable populations from Ruaha have been isolated for a long time too. Thus, restoration of corridors may facilitate the gene flow between these ecosystems, but more detailed genetic research needs to be conducted to cover the entire Selous ecosystem and Mozambique populations to reveal the most important corridors. A wildlife corridor between Selous Game Reserve and Niassa Reserve in Mozambique was established in 1998 (Baldus & Hahn, 2009). This corridor may be even more important for elephants and other wildlife than the Ruaha‐Selous corridors. Genetic evidence from sable antelope and lions supports that their Selous populations are genetically more closely related to Southern Africa than Ruaha. Our elephant samples came from a small subarea of these large Ruaha and Selous ecosystems, and there could be different subpopulations further south and west that we did not sample. Therefore, it is possible that we missed evidence of more significant gene exchange between Ruaha and Selous. Future studies should be conducted to cover the entire Selous ecosystem and Niassa Game Reserve in Mozambique.

There are geographic elements, such as the Rift valley and the chain of Eastern Arc mountains that can impede migration/gene flow of elephants between protected areas. However, there have been natural corridors that have been used historically by elephants and other species. Unfortunately, these natural corridors are often associated with streams/rivers which make these areas suitable for human activities and settlements. For example, agricultural expansion and human immigration in the Kilombero Valley are one of the significant challenges facing wildlife corridors in that area of southern Tanzania. Conservationists and policy makers are faced with difficult choices and issues to restore migratory corridors where there are human activities. We support the approach suggested by Jones et al. (2012) for the restoration of wildlife corridors which consists of opening new Wildlife Management Areas in which communities are active participants and benefit from corridor protection, and purchasing land from private land owners. Kioko et al. (2013) suggested that the area between Manyara Ranch and Lake Manyara should be included as a Wildlife Management Area to facilitate connectivity between Manyara Ranch and Lake Manyara elephants, which seem to be isolated. Establishment of Wildlife Management Areas (WMA) in Tanzania has increased habitats and protection of wildlife species. For example, Lee (2018) documented significantly higher densities of several wild ungulate species and lower frequencies of domestic ungulates in the Burunge WMA compared with adjacent village lands. Burunge WMA is within the Tarangire‐Manyara ecosystem and provides potential connectivity between Tarangire and Lake Manyara National Parks.

Elephants retain memories of important habitat and resources information, and recalling these memories are important for survival of multigenerational matriarchal family groups (McComb, Moss, Durant, Baker, & Sayialel, 2001). Thus, protecting wildlife corridors could reduce the extent of human‐elephant conflicts in Tanzania, when elephants seek to access historically important areas that have since become inaccessible by human activities. Development of infrastructure such as roads is inevitable for social and economic development. Roads may act as significant barriers for movement of species (Gaynor et al., 2018). In the long run, constructing wildlife overpass crossings, for example, across the Arusha‐Babati road, may be the best option to increase genetic connectivity between subpopulations in northern Tanzania although the larger threat is land conversion through agriculture. Traditional protected area systems have long been considered the most effective way of protecting wildlife in Tanzania. Indeed, most wildlife species are found within these protected areas. However, the role of more human‐dominated landscapes, especially those adjacent to protected areas, is essential for dispersal areas for large mammals like wildebeest and elephants. Here, we want to emphasize other categories of protected areas which can accommodate both human use and wildlife conservation, such as WMAs and wildlife ranches, like Manyara Ranch.

There is a need to recognize the importance of conserving wildlife in human‐dominated areas (Ogutu, Kuloba, Piepho, & Kanga, 2017). Formal protected areas such as national parks and game reserves are not enough for the conservation of far‐ranging species such as elephants. Communities that have provided some portion of their land have helped provide habitats for many species and villagers have started benefiting financially from conservation projects. Wildlife Management Areas, for example, have been a source of income for some villages. These funds can be used for social development projects such as building schools, health centers, and water supply. However, a proper land use plan and community rights of occupancy should be considered before implementing these projects. We recommend village leaders to consult organizations such as the Ujamaa Community Resource Team (UCRT) which has empowered many villages in northern Tanzania to secure rights of their natural resources and land. UCRT also help these communities by representing their land rights, advocating on their behalf to local and national government, and securing legal ownership of their traditional lands (http://www.ujamaa‐crt.org). Indeed, the future of wildlife conservation in Tanzania, particularly for far‐ranging species, and in the face of increasing isolation of protected areas, is reliant upon participation of communities whose livelihoods depend on these same areas. Every effort must be made to ensure it is a mutually beneficial arrangement as recommended in Table 4.

## CONFLICT OF INTEREST

None declared.

## AUTHOR CONTRIBUTION

**George G. Lohay:** Conceptualization (lead); Data curation (lead); Formal analysis (lead); Funding acquisition (equal); Investigation (equal); Methodology (lead); Project administration (equal); Resources (equal); Supervision (supporting); Validation (lead); Visualization (equal); Writing‐original draft (lead); Writing‐review & editing (lead). **Thomas Casey Weathers:** Conceptualization (supporting); Data curation (supporting); Formal analysis (equal); Investigation (equal); Methodology (equal); Validation (supporting); Writing‐review & editing (supporting). **Anna B. Estes:** Conceptualization (supporting); Formal analysis (supporting); Funding acquisition (supporting); Investigation (supporting); Methodology (supporting); Resources (supporting); Supervision (supporting); Validation (equal); Writing‐original draft (equal); Writing‐review & editing (equal). **Barbara C. McGrath:** Conceptualization (supporting); Data curation (equal); Formal analysis (equal); Funding acquisition (supporting); Investigation (supporting); Methodology (equal); Project administration (supporting); Resources (lead); Supervision (equal); Validation (equal); Writing‐original draft (equal); Writing‐review & editing (equal). **Douglas R. Cavener:** Conceptualization (lead); Data curation (equal); Formal analysis (lead); Funding acquisition (lead); Investigation (supporting); Methodology (supporting); Project administration (lead); Resources (lead); Software (equal); Supervision (lead); Validation (lead); Visualization (equal); Writing‐original draft (supporting); Writing‐review & editing (equal).

## Supporting information

Appendix S1‐S4

## Data Availability

DNA sequences have been deposited in GeneBank (accession numbers MN194226–MN194258) and microsatellite genotypes and sampling locations archived in Dryad DOI (https://doi.org/10.5061/dryad.zs7h44j4m).
