# Supplementary material for: Genetic connectivity and population structure of African savanna elephants (Loxodonta africana) in Tanzania
Source: Ecol Evol. 2020 Oct 9;10(20):11069–89. doi: 10.1002/ece3.6728 (PMC7593188; doi:10.1002/ece3.6728)
Supplement: Supplementary file 1 — Appendix S1‐S4 [file ECE3-10-11069-s001.docx]

**Appendices**

**Appendix S1**. Characterization of 11 microsatellite loci for the African Savanna elephants.

| **Primer** | **Original Source** | **Primer Sequence 5’-3’** | **Dye** | **Panel** | **Fragment length** | **Ta ºC** |
| --- | --- | --- | --- | --- | --- | --- |
| LA5-F | Eggert et al. (2000) | GGGCAGCCTCCTTGTTTT | NED | 3 | 139-155 | 58 |
| LA5-R |  | CTGCTTCTTTCATGCCAATG |  |  |  | 58 |
| FH60-F | Comstock et al. (2000) | CAAGAAGCTTTGGGATTGGG | VIC | 3 | 143-163 | 58 |
| FH60-R |  | CCTGCAGCTCAGAACACCTG |  |  |  | 58 |
| LaT24-F | Archie et al. 2003 | AAGTTGAGAGATCAGCAAAGCA | VIC | 1 | 124-264 | 58 |
| LaT24-R |  | GATGTTCAGTCCTTCCTTAGCA |  |  |  | 58 |
| LA6-F | Eggert et al. (2000) | AAAATTGACCCAACGGCTC | VIC | 1 | 145-177 | 58 |
| LA6-R |  | TCACGTAACCACTGCGCTA |  |  |  | 58 |
| FH19-F | Comstock et al. (2000) | GAAGCTCATGGTCAAGGTCAC | 6-FAM | 4 | 185-207 | 58 |
| FH19-R |  | CTGCATACTCATCGAAGTCACC |  |  |  | 58 |
| FH67-F | Comstock et al. (2000) | GCTTCTCTAGAAATGTGTATGC | NED | 1 | 88-110 | 55 |
| FH67-R |  | GGCGTATAGGATAGTTCCAC |  |  |  | 55 |
| LafMS02-F | Nyakaana et al. (2005) | GAAACCACAACTTGAAGGG | NED | 2 | 136-168 | 55 |
| LafMS02-R |  | TCGCTTGTAAGAAGGCGTG |  |  |  | 55 |
| FH48-F | Comstock et al. (2000) | GAGTCTCCATAATCAAGAGCG | NED | 2 | 166-182 | 55 |
| FH48-R |  | CCTCCCTGGAATCTGTACAG |  |  |  | 55 |
| LaT08-F | Archie et al. 2003 | ATGGACAGGCAGAAAGATTT | 6-FAM | 1 | 177-230 | 55 |
| LaT08-R |  | TCCCAATAACAGGATAGCATT- |  |  |  | 55 |
| LaT06-F | Archie et al. 2003 | AGCCAGGCACATTAAGTGT | 6-FAM | 2 | 270-398 | 55 |
| LaT06-R |  | CTCCTAGAAAAGGTTACCAC |  |  |  | 55 |
| LaT13-F | Archie et al. 2003 | AGCTTCTGTAGGCTCTGA | NED | 2 | 216-272 | 55 |
| LaT13-R |  | ACTCGATAAACAGTGTTGA |  |  |  | 55 |


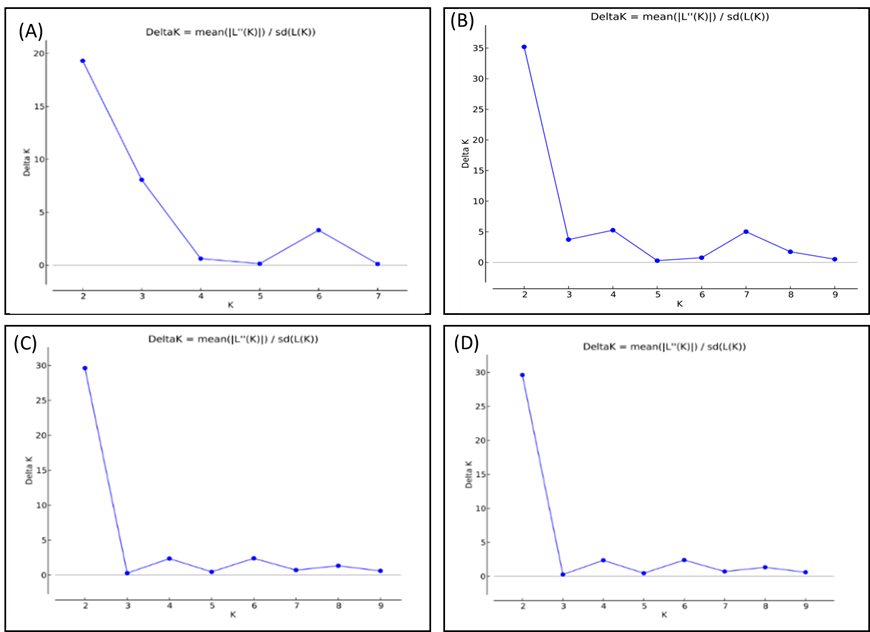

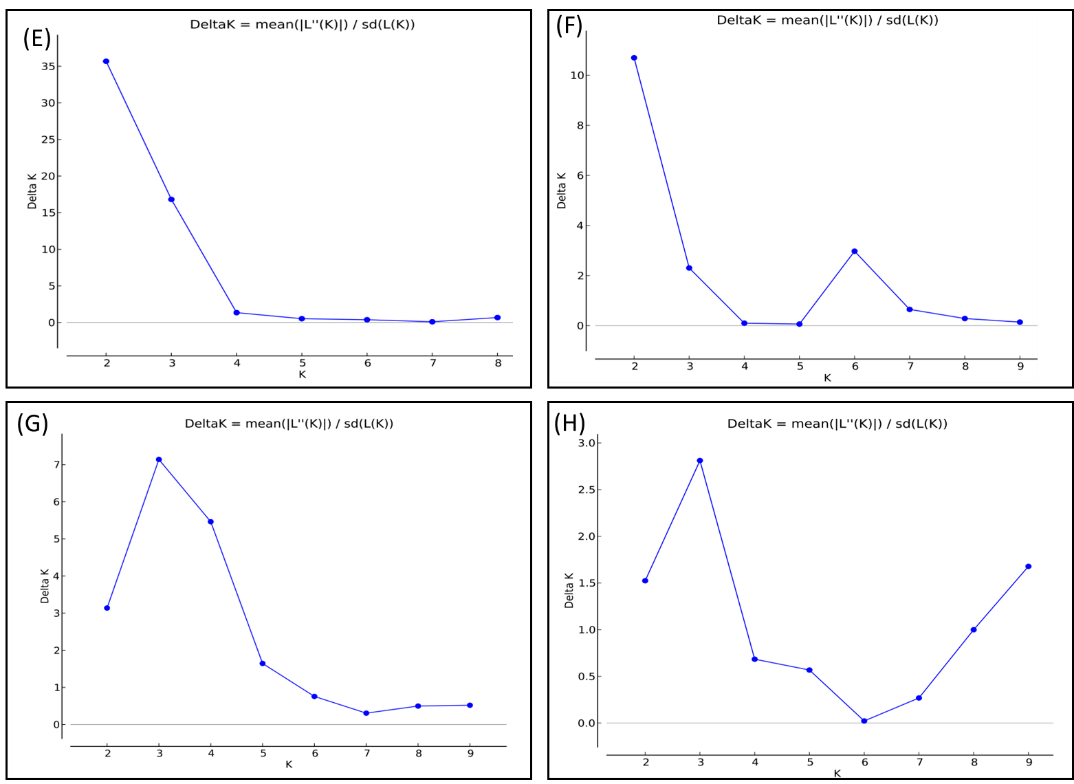


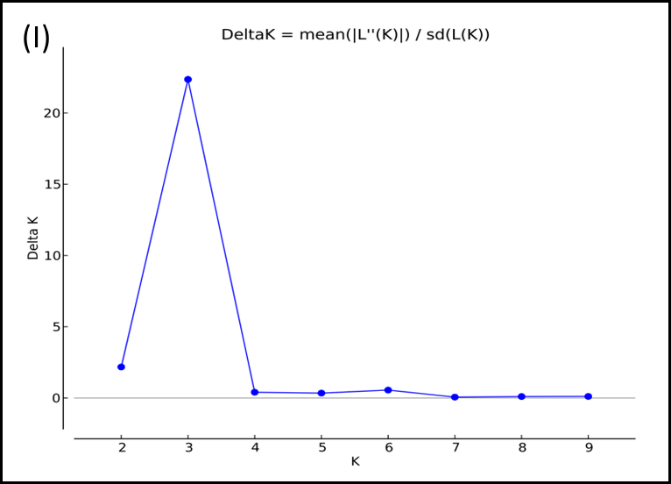


**Appendix S2**. Supporting Δ*K* plots used to estimate African savanna elephant population clusters inferred from hierarchical analysis using STRUCTURE.

**Appendix S3**. Distribution of 32 mtDNA haplotypes for African elephants in northern and southern Tanzania.

|  | Haplotype | NSE | SSE | NCA | MAR | TNP | RNP | SGR | Total |
| --- | --- | --- | --- | --- | --- | --- | --- | --- | --- |
| 1 | ECSE09* | 176 | 33 | 29 | 10 | 3 |  |  | 251 |
| 2 | ECSE11* | 2 | 2 | 6 |  | 4 |  |  | 14 |
| 3 | ECGR02 | 3 |  |  |  |  |  |  | 3 |
| 4 | ECGR01 | 1 |  |  |  |  |  |  | 1 |
| 5 | ECSE10 | 2 | 2 |  |  |  |  |  | 4 |
| 6 | ECLM06 |  |  |  | 1 |  |  |  | 1 |
| 7 | ECNG03* | 2 | 2 | 2 | 9 |  |  |  | 15 |
| 8 | ECSE08 | 1 |  |  |  |  |  |  | 1 |
| 9 | ECLM03 |  |  |  | 4 |  |  |  | 4 |
| 10 | ECSE07 | 1 |  |  |  |  | 1 |  | 2 |
| 11 | ECMG01 |  | 1 |  |  |  |  |  | 1 |
| 12 | SWSE03 | 2 | 1 |  |  |  |  |  | 3 |
| 13 | SWTA01* | 10 | 14 | 27 | 32 | 35 | 21 |  | 139 |
| 14 | SWMR02 |  |  |  | 13 |  |  |  | 13 |
| 15 | SWMR03 |  | 3 | 1 | 2 |  |  |  | 6 |
| 16 | SWLM02 |  |  |  | 2 |  |  |  | 2 |
| 17 | SWSE04* | 7 | 6 | 7 |  |  |  |  | 20 |
| 18 | SWSE02* | 9 | 1 |  |  |  |  |  | 10 |
| 19 | SWMR01 |  | 4 |  | 1 |  |  |  | 5 |
| 20 | SSRU01 |  |  |  |  |  | 3 |  | 3 |
| 21 | SSNG01* | 1 | 4 | 6 | 9 |  |  | 4 | 24 |
| 22 | SSSE01 | 3 | 2 |  |  |  |  |  | 5 |
| 23 | SSNG02 |  |  | 4 | 1 |  |  | 1 | 6 |
| 24 | SSSG01 |  |  |  |  |  |  | 5 | 5 |
| 25 | SSSG02 |  |  |  |  |  |  | 5 | 5 |
| 26 | SSSG03 |  |  |  |  |  |  | 4 | 4 |
| 27 | SSSG04 |  |  |  |  |  |  | 1 | 1 |
| 28 | SSSG05 |  |  |  |  |  |  | 4 | 4 |
| 29 | SSSG06 |  |  |  |  |  |  | 1 | 1 |
| 30 | SSSG07 |  |  |  |  |  |  | 1 | 1 |
| 31 | SSSG08 |  |  |  |  |  |  | 1 | 1 |
| 32 | SSSG09 |  |  |  |  |  |  | 3 | 3 |
|  | **Total** | **220** | **75** | **82** | **84** | **42** | **25** | **30** | **558** |

Haplotype names have two parts: subclade names (EC=East-central, SW=Savanna-wide and SS=Southeast-savanna). Haplotypes identified in previous studies are indicated with asterisks, the rest are new haplotypes unique to this study. Abbreviations for each of the sampling locations are indicated in Table 1.

**Appendix S4**. Number of elephant fecal samples collected from each sampling locations in Tanzania

| Sampling locations | Samples collected | Samples genotyped | Genotyped samples (microsatellites) | Sequenced samples (mtDNA) |
| --- | --- | --- | --- | --- |
| Northern Serengeti (NSE) | 247 | 210 | 212 | 220 |
| Southern Serengeti (SSE) | 123 | 121 | 112 | 75 |
| Ngorongoro Conservation Area (NCA) | 126 | 114 | 104 | 82 |
| Manyara (MAR) | 138 | 103 | 98 | 84 |
| Tarangire National Park (TNP) | 67 | 67 | 64 | 42 |
| Ruaha National Park (RNP) | 49 | 49 | 48 | 25 |
| Selous Game Reserve (SGR) | 50 | 50 | 50 | 30 |
| Total | **800** | **714** | **688** | **558** |
